# Regional genome transcriptional response of adult mouse brain to hypoxia

**DOI:** 10.1186/1471-2164-12-499

**Published:** 2011-10-11

**Authors:** Huichun Xu, Aigang Lu, Frank R Sharp

**Affiliations:** 1 Center for Research on Genomics and Global Health, National Human Genome Research Institute, National Institutes of Health, 12 South Drive, Bethesda, MD 20892-5635, USA (Current; 2 Department of Neurology and M.I.N.D. Institute, University of California at Davis Medical Center, 2805 50th Street, Sacramento, CA 95817, USA; 3 Department of Neurology, Vontz Center for Molecular Studies, University of Cincinnati, Cincinnati, OH 45267-0532, USA

## Abstract

**Background:**

Since normal brain function depends upon continuous oxygen delivery and short periods of hypoxia can precondition the brain against subsequent ischemia, this study examined the effects of brief hypoxia on the whole genome transcriptional response in adult mouse brain.

**Result:**

Pronounced changes of gene expression occurred after 3 hours of hypoxia (8% O_2_) and after 1 hour of re-oxygenation in all brain regions. The hypoxia-responsive genes were predominantly up-regulated in hindbrain and predominantly down-regulated in forebrain - possibly to support hindbrain survival functions at the expense of forebrain cognitive functions. The up-regulated genes had a significant role in cell survival and involved both shared and unshared signaling pathways among different brain regions. Up-regulation of transcriptional signaling including hypoxia inducible factor, insulin growth factor (IGF), the vitamin D3 receptor/retinoid X nuclear receptor, and glucocorticoid signaling was common to many brain regions. However, many of the hypoxia-regulated target genes were specific for one or a few brain regions. Cerebellum, for example, had 1241 transcripts regulated by hypoxia only in cerebellum but not in hippocampus; and, 642 (54%) had at least one hepatic nuclear receptor 4A (HNF4A) binding site and 381 had at least two HNF4A binding sites in their promoters. The data point to HNF4A as a major hypoxia-responsive transcription factor in cerebellum in addition to its known role in regulating erythropoietin transcription. The genes unique to hindbrain may play critical roles in survival during hypoxia.

**Conclusion:**

Differences of forebrain and hindbrain hypoxia-responsive genes may relate to suppression of forebrain cognitive functions and activation of hindbrain survival functions, which may coordinately mediate the neuroprotection afforded by hypoxia preconditioning.

## Background

Given that brain depends upon rapid and continuous delivery of oxygen to maintain normal function and survival, it may have developed unique molecular responses to hypoxia as compared to other organs and organisms. In spite of the crucial role of oxygen in normal brain function, there have been relatively few studies of brain hypoxia at the whole genome level. Previous studies using early-generation microarrays found changes of expression in whole brain of neonatal rat (34 genes up-regulated, 42 genes down regulated) and in cerebral cortex of adult mice (29 genes up regulated) [1,2]. Thus, this study was undertaken in part to examine the brain hypoxia response at the whole genome level *in vivo*.

One of the major goals of this study, however, was to define the regional, whole genome changes of gene expression that could account for the phenomenon of "hypoxia preconditioning" (HP)[3-7]. In the first described brain HP model, exposure of neonatal rats to 3 hours of 8% oxygen almost completely protected against hypoxia-ischemia induced infarction [3]. In adult mouse brain, hypoxia preconditioning 24 to 48 hours in advance can reduce infarct volume up to 64% [6,8]. Moreover, the protection provided by HP can last 5-8 weeks [9,10]. Thus, HP may offer an important platform for the discovery of neuroprotective targets. Though extensively studied, the precise mechanisms accounting for HP remain unknown. Since HP requires new RNA and protein synthesis, we set out to study the whole genome transcriptional response to HP in the adult C57BL/6 mouse brain using Affymetrix Expression Arrays (> 39,000 transcripts) [7,11,12]. The dynamic gene expression changes were assessed over the following 24 hours after HP- a time by which the brain would be protected against focal ischemia [7]. Moreover, the expression changes were assessed on a region by region basis: the regional differences of the brain regarding its expression response to hypoxia/HP have not been studied systemically. It is known that certain brain regions and cells, such as hippocampal pyramidal neurons, striatal medium spiny neurons and cerebellar Purkinje neurons, are quite vulnerable to hypoxia/ischemia whereas others can be relatively resistant[13,14]. It is still not clear whether hypoxia/HP responses could have predominantly engaged common or different mechanisms in different regions. One previous study, using a neonatal rat hypoxia-preconditioning model, showed differential molecular responses in neocortex, hippocampus, striatum and thalamus, even though HP produced similar protection in these regions [15]. Thus, our current study further examined the regional response to HP systemically at the genomic level to determine whether there were common as well as region-specific responses to hypoxia (8% O_2_) in the brain.

Our study examined both time- and region-dependent transcriptional responses induced by HP in the adult mouse brain. Besides hypoxia inducible factor (HIF), various nuclear receptor transcription factors have been found to play important roles in regulating both the region-independent and region-specific gene expression responses to HP. Different brain region exhibited differential but coordinated responses: in the forebrain, down regulation of gene expression was predominant during peak response period while the hindbrain, especially the cerebellum, demonstrated predominant up-regulation responses to HP. Surprisingly, the cerebellum demonstrated the most profound gene expression response among all regions and may play a pivotal role in the protective effect of HP *in vivo*.

## Results

### Many hypoxia regulated gene products are in the nucleus

A one-way ANOVA, corrected for multiple comparisons using a False Discovery Rate of 5%, yielded 2,324 transcripts that changed 1.5 fold or more in at least one brain region after HP treatment in adult C57BL/6 mice. Many (~40%) of these 8% O_2 _hypoxia-regulated genes have unknown functions. For the well annotated hypoxia-regulated transcripts, almost half of the gene products are located in the nucleus (48.2%) (Additional file 1 figure S1A). Of these, many were transcriptional regulators (34%) (Additional file 1 figure S1B).

### Hypoxia-inducible transcription factor (HIF)

**Figure 1:**
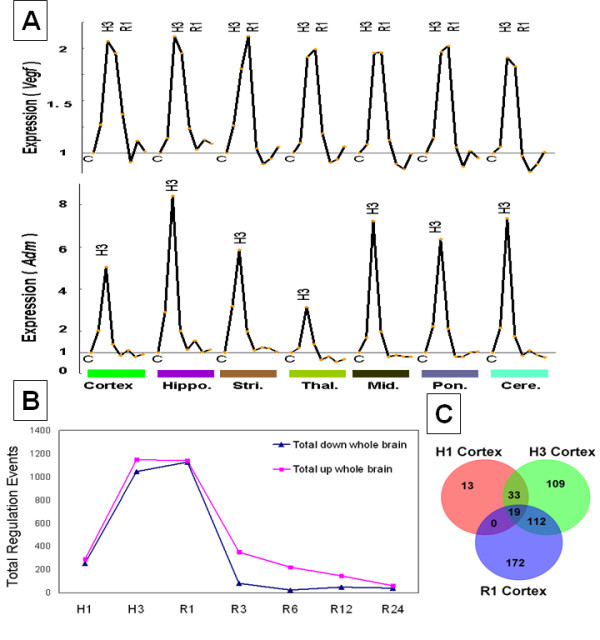
**Time dependent gene expression changes induced by hypoxia**. **(A) **Time course of expression of selected HIF-1 alpha target genes in all brain regions studied derived from the microarray experiments. ***Adm*: **Adrenomedullin. ***Vegfa*: **Vascular endothelial growth factor A. The y-axis is the expression value (normalized to the sham controls and log-transformed). The x-axis shows the time course of gene expression in the following order: C, control; H1, 1 hour of hypoxia; H3, 3 hours of hypoxia; R1, 1 hour after reoxygenation; R3, 3 hours after reoxygenation; R6, 6 hours after reoxygenation; R12, 12 hours after reoxygenation; R24, 24 hours after reoxygenation. Regions: cerebral cortex (Cortex), hippocampus (Hippo.), striatum (Stri.), thalamus (Thal.), midbrain (Mid.), pons and medulla (Pon.), cerebellum (Cere.). **(B)**. Total number of up-regulated transcripts and total number of down-regulated transcripts in the entire brain. The y-axis is the number of up-regulated transcripts (red line) or down-regulated transcripts (blue line) summed across all brain regions at each time point. The x-axis shows the time courses following hypoxia. **(C)**. Venn diagram of time-dependent gene expression changes in cerebral cortex from H1 through R1. These genes were derived by performing a one-way ANOVA with a False Discovery Rate of 5% and with a fold change of ≥ 1.5 or greater.

Of the 70 verified HIF-1α target genes [16], 17 changed expression in brain following hypoxia (Additional file 2 table S1). Five of these genes--*Adm, Cdkn1a, Ddit4, Ets1, and Vegfa*--had a similar time course of expression changes in all brain regions, although the magnitudes of their expression were not the same across different regions (Figure 1A, Additional file 1 figure S2). The other 12 HIF-1α target genes were differentially regulated in different brain regions (Additional file 2 table S1). Though there were more than 2,000 genes regulated following hypoxia, only 92 changed expression in all of the brain regions. Notably, 48% of these 92 genes (38 genes) were identified as potential HIF target genes by promoter analysis using the Genomatix Gene2promoter analysis tool (Additional file 2 table S2).

### Time course of gene expression regulation

**Figure 2:**
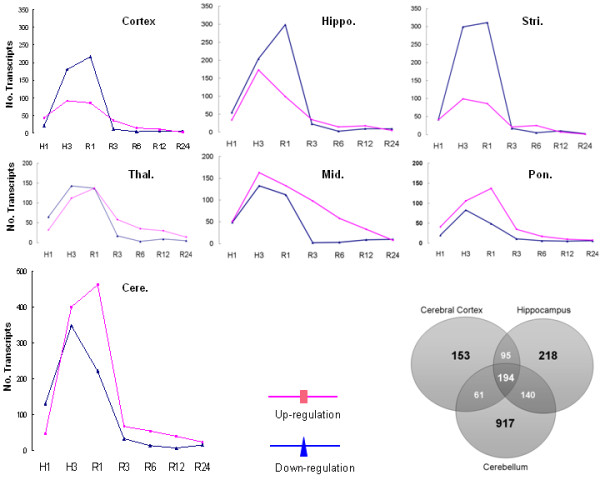
**Regional differences in expression responses to hypoxia**. Total number of up-regulated transcripts (red line, y-axis) was compared to the number of down-regulated transcripts (blue line, y-axis) in each brain region as a function of time (x-axis). **Insert: **Venn diagram of numbers of hypoxia-regulated transcripts in cerebral cortex (Cortex), hippocampus (Hippo.), and cerebellum during the whole time course investigated using a one way ANOVA, FDR < 0.05 and fold change > 1.5.

The total numbers of genes that had increased or decreased expression at each time point were summed for the entire brain (Figure 1B). The greatest numbers of regulated genes occurred immediately after the 3 hours of hypoxia and at one hour after re-oxygenation. Thereafter, the number of regulated genes decreased over time, with only a few regulated at 24 hours after the hypoxia exposure (Figure 1B). This evolving pattern of gene expression regulation over time also held true for each individual brain region (Figure 2). For a given brain region there were many more genes regulated at 3 hours of hypoxia compared to 1 hour of hypoxia, and many different genes regulated at 1 hour of re-oxygenation compared to 3 hours of hypoxia (Figure 1C). Thus, there were relatively few genes that were commonly regulated at two or more time points in a given brain region (Figure 1C).

### Region-dependent gene expression changes

As noted above, only 92 of the 2,324 transcripts regulated by hypoxia were regulated in all of the brain regions investigated. Thus, the hypoxia-regulated genes differed between brain regions (Figure 2, venn diagram) as did the total number of regulated genes. The pons and medulla had the fewest number of regulated genes (330 transcripts with a 1.5-fold threshold) and cerebellum had the most (1312 transcripts with a 1.5-fold threshold) (Figure 2). For each region there were up- and down-regulated genes though down regulated genes were more numerous in the forebrain (Figure 2, cortex, hippocampus, striatum, and thalamus) whereas up-regulated genes predominated in the midbrain, medulla/pons, and particularly, cerebellum (Figure 2). However, the total numbers of up- and down-regulated genes in the brain as a whole are fairly comparable to each other at each time point (Figure 1B).

### Gene expression changes in the forebrain related to cell survival

There were generally many more down-regulated genes than up-regulated genes in forebrain regions, a finding similar to that reported for ischemic-preconditioning [17]. A given gene was usually either down- or up-regulated during the entire time course with very few exceptions: only 3 out of 503 genes in the cortex, 12 out 647 genes in the hippocampus, and 8 out 704 genes in the striatum showed expression changes in both directions during the 24 hour period. This means that hypoxia-regulated genes can be simply divided into two classes: up-regulated or down-regulated.

**Figure 3:**
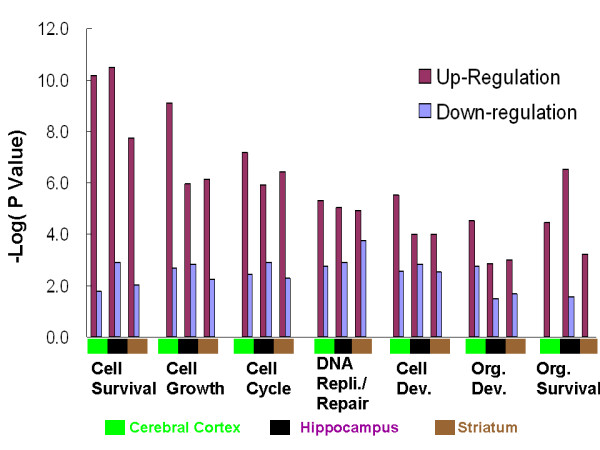
**Molecular functions associated with up-regulated genes and with down-regulated genes within each forebrain region**. The x-axis shows the categories of molecular functions related to cell death/survival. The individual forebrain regions are marked below the x-axis with colored squares: red, cerebral cortex; blue, hippocampus; purple, striatum. The y-axis is the absolute value of the log transformed P value, which means that a smaller P value has a larger positive value on the y-axis. The value 1.3 on the y-axis is equivalent to a P value of 0.05. The log transformed P value for the functions associated with up-regulated genes is colored dark red, while the value for down-regulated genes is colored light blue. Dev.: Development; Org.: Organism; Repli.: Replication.

We then compared the overall molecular functions of the up- and down-regulated genes in the forebrain using the Ingenuity Knowledge Database. To minimize the potential bias caused by an arbitrary fold change filter, a minimum threshold of 1.2-fold was used for all pathway analyses. Within each forebrain region, genes involved in regulation of overall organismal survival and development, cell death and survival, and cellular growth and proliferation were significantly over-represented in the up-regulated genes compared to the down-regulated genes (Figure 3). Similar findings were obtained for all three forebrain regions even when different fold change thresholds were used. This result was further confirmed by interaction network analysis, which was used to construct the most prominent clusters of genes in each of the forebrain regions based on known molecular interactions in the literature. The top networks of genes formed by the up-regulated genes were associated with cell death/survival and proliferation in each of the forebrain regions (Additional file 2 table S3).

Since cerebral cortex, hippocampus and striatum appear to respond similarly to hypoxia in terms of predominance of a decrease of gene expression, we searched for common signaling pathways among them. Using a 1.2-fold threshold, 314 transcripts were found to be regulated in all three regions in the forebrain. A two-way ANOVA analysis (regions and time courses without FDR correction, p<0.05) on these 314 genes further excluded 14 genes which showed region-dependent expression changes among the forebrain regions. This left 300 HP-regulated transcripts that had a consistent temporal pattern of expression changes across cortex, hippocampus and striatum. This was confirmed using an unsupervised cluster analysis: the expression profiles at the same time points from different regions were similar to one another and therefore clustered together compared to those from neighboring time points (Additional file 1 figure S3).

Since the up-regulated genes in the forebrain had more significant roles in regulating cell survival than down-regulated genes (Figure 3), we focused our signal pathway analysis on the common up-regulated genes only (87 out of the 314 transcripts, the top cluster of genes shown in Additional file 1 figure S3 and table S4). Many signaling pathways were activated in response to the hypoxia, and no single pathway or molecule can account for the entire observed changes (Table 1). The complexity of the gene/pathway responses following hypoxia is demonstrated by the interaction network formed by selected genes from the 87 up-regulated transcripts (Additional file 1 figure S4).

**Table 1:** Signaling pathways significantly associated with commonly up-regulated genes in all the forebrain regions after HP.

Level	Signal Classification	Pathway	P value	Molecules*
System level	Hormone	Glucocorticoid Receptor Signaling	0.006	NFKBIA, FKBP5, CDKN1A, TSC22D3, SGK1
	
	Hypoxia signaling	Hypoxia signaling (Cardiovascular System)	0.016	NFKBIA, VEGFA

Tissue level	Synapses	Clathrin-mediated Endocytosis	0.076	VEGFA, AP2A2
	
	Development	Axonal Guidance/Ephrin Receptor Signal	0.093	VEGFA, GNA13

Cell level	Growth factors	Insulin/IGF-1 Receptor Signaling	0.054	PPP1R3C, SGK1, CYR61
		
		Neuregulin/EGF Signaling	0.028	ERRFI1, TMEFF2
	
	Survival regulation	VDR/RXR Activation	0.023	KLF4, CDKN1A
		
		p53 Signaling	0.026	CDKN1A, TP53INP1
	
	Kinase cascade	PI3K/AKT Signaling	0.046	NFKBIA, CDKN1A
		
		ERK/MAPK Signaling	0.016	H3F3B, PPP1R3C, H3F3A

The significance was determined by Fisher's exact test(P<0.1). Pathways with the same gene hits or with closely related genes were combined. The significance of the combined pathway was determined by the smallest P value available before the combining.* Details of the genes can be found in Additional file 2 Table S2.

The above pathway analyses suggested that a number of transcription regulators likely play important roles in up-regulating gene expression responses to hypoxia, including HIF, glucocorticoid receptors and insulin like growth factor (Table 1). To directly address which transcription factors might be the most important, we performed promoter analysis for the genes that were induced after just 1 hour of hypoxia-This time point was chosen because 1 h of preconditioning hypoxia is sufficient to produce a protective effect comparable to that induced by 3 hr or 6 hr of preconditioning hypoxia [8]. Among the 87 up-regulated transcripts showing consistent time courses of expression across the three forebrain regions, 37 of these transcripts were up-regulated early at one hour of hypoxia. Promoter analyses on these genes (Genomatix Software) identified fourteen transcription factor families that could have initiated expression changes of these genes (Table 2). Transcription factor families V$GREF (Glucocorticoid Responsive and related Elements) and V$HIFF (HIF) were once again present in the list, which is consistent with the above pathways analysis (Table 1). Moreover, the gene for one of the candidate transcription factors, *Klf4*, was itself up-regulated at 1 hr after the onset of preconditioning hypoxia (Additional file 1 figure S5).

**Table 2:** Candidate transcription factor families responsible for the early transcriptional up-regulation events in the forebrain at 1 hr after HP.

Name	Transcription Factor Families	GO function annotation	P Value	Representative members	Preferentially associated with CNS
V$NFKB	Nuclear factor kappa B/c-rel	apoptosis, oxidative stress	0.002	Nfkb	

V$CP2F	CP2-erythrocyte Factor related to drosophila Elf1	steroid biosynthetic process	0.003	Cp2	

V$YBXF	Y-box binding transcription factors	negative regulation of apoptosis, response to cold	0.003	Csda	

V$CTCF	CTCF and BORIS gene family transcriptional regulators	DNA methylation	0.005	Ctcf	

V$GLIF	GLI zinc finger family	CNS development, negative regulation of cell proliferation, neurogenesis	0.005	Gli1/Gli3	Yes

V$EBOX	E-box binding factors	apoptosis, cell cycle arrest, cell proliferation, ER stress response, regulation of glycolysis	0.008	Nmyc-1, Myc	Ubiquitous

V$AHRR	AHR-arnt heterodimers and AHR-related factors	apoptosis, cell cycle, nervous system development, response to stress	0.010	AHR/ARNT dimers	

V$AP2F	Activator protein 2	cell-cell signaling, nervous system development	0.010	AP2	Ubiquitous

V$NOLF	Neuron-specific-olfactory factor	positive regulation of transcription	0.011	Ebf1	Yes

V$GKLF	Gut-enriched Krueppel like binding factor	mesodermal cell fate determination, negative regulation of cell proliferation	0.029	Klf4	

V$HESF	Vertebrate homologues of enhancer of split complex	Notch signaling pathway, cell proliferation, circadian clock, negative regulation of neuron differentiation, vasculogenesis	0.034	Hes1-6	Yes

V$MZF1	Myeloid zinc finger 1 factors	N/A	0.034	Mzf1	Ubiquitous

V$HIFF	Hypoxia inducible factor, bHLH/PAS protein family	CNS development, oxygen homeostasis, circadian rhythm, response to temperature stimulus	0.042	HIF-1α, HIF-2α	Ubiquitous

V$GREF	Glucocorticoid responsive and related elements	cell growth, cell proliferation, glucocorticoid receptor signaling pathway	0.047	Nr3c1	Ubiquitous

Since some or many of the changes of gene expression described here may play a role in producing hypoxia-preconditioning and protecting brain against subsequent stroke, we examined the hypothesis that hypoxia may have modulated many of the same genes as were modulated by ischemic stroke. We therefore compared the hypoxia-regulated genes reported here to stroke-regulated genes identified by recent microarray studies with Affymetrix GeneChip^®^. One study from our group examined the adult Sprague-Dawley rat brain following a permanent middle cerebral artery occlusion procedure (MCAO) with Affymetrix rat U34A array [18]. The other study, conducted by Sarabi et al., surveyed the same C57BL/6J adult mice brain following transient MCAO procedure with the same Affymetrix mouse MU430_2 array used in our current study [19]. In both studies gene expression was assessed in cerebral cortex within 24 hours after the stroke. 575 rat transcripts on the rat U34A array (8,799 transcripts assessed) were regulated at least 1.5 fold in the periinfarction cortex by acute ischemic stroke, which were homologous to 1000 transcripts on our mouse MU430_2 array. Among the 1000 stroke-regulated transcripts, only 12 transcripts were also regulated by hypoxia according to our current study (Additional file 2 table S5). Sarabi et al. identified 265 transcripts that changed expression following stroke [19], but only 13 of these were also regulated by hypoxia in our current study (Additional file 2 table S5). Therefore, even though hypoxia changed expression of 503 transcripts in the mouse cerebral cortex, less than 5% (24 transcripts) were also regulated by ischemia.

### Unique response of the cerebellum to hypoxia compared to hippocampus

Not only did cerebellum exhibit the most expression changes following HP among all brain regions (1312 transcripts in cerebellum vs. 647 transcripts in hippocampus with a 1.5-fold threshold), it had the largest number of up-regulated genes. We therefore explored the uniqueness of hindbrain structure cerebellum's gene expression response to HP in comparison with the forebrain structure hippocampus because they are comparable in many aspects. They each have a particularly vulnerable cell type: pyramidal cells in hippocampus and Purkinje cells in cerebellum; structurally they share similar grey matter and white matter divisions; and both are relatively primitive brain structures that originated from the archipallium.

**Figure 4:**
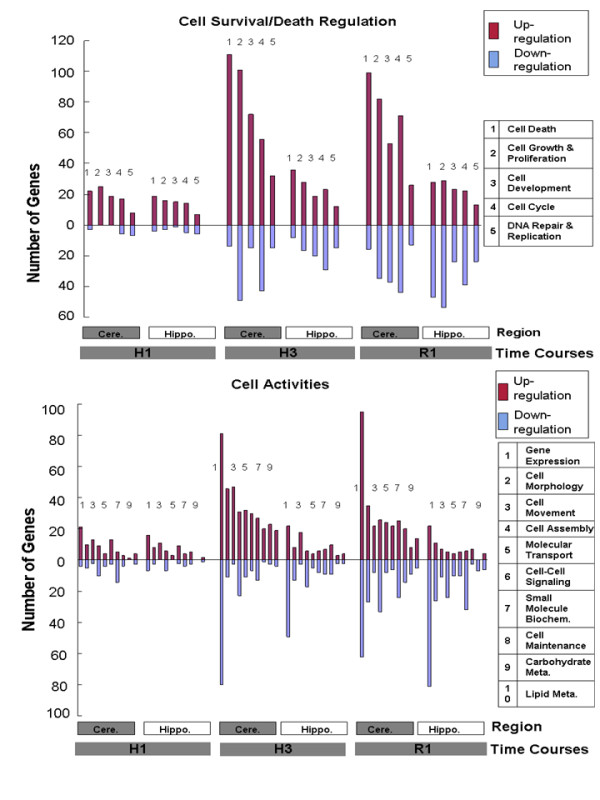
**Functional comparison of genes regulated by hypoxia in cerebellum versus hippocampu**. **(A)**. Comparison of the number of genes up- or down-regulated by hypoxia that have functions related to cell death/survival in the cerebellum and in the hippocampus. **(B)**. Comparison of the number of genes up- or down-regulated by hypoxia associated with selected cellular functions in cerebellum and in hippocampus. The side-by-side comparison between cerebellum and hippocampus was made at three time points: immediately after 1 hr of hypoxia (H1), immediately after 3 hr of hypoxia (H3), and 1 hr after reoxygenation (R1). Y-axis shows the number of up (dark red bar,) or down (blue bar) regulated genes associated with each category of molecular functions in each region. Different categories of functions were indexed to the right of the figure. Biochem.: Biochemistry; Meta.: Metabolism.

Molecular functions of the hypoxia regulated genes were explored in each brain region using Ingenuity software. Analyses were performed on the genes regulated at one hour of hypoxia (H1), three hours of hypoxia (H3) and one hour of re-oxygenation (R1) since the largest numbers of genes were regulated at these times in all brain regions (Figure 2). Hypoxia-regulated genes that differed between cerebellum and hippocampus fell into two broad categories: cell survival, growth and proliferation (Figure 4A); and cell activities, including cell morphology, cell movement, cell maintenance, cell to cell signaling, molecular transport, metabolism and gene expression, which collectively reflect the overall vibrancy of the cell (Figure 4B). Similar numbers of genes were moderately up-regulated in cerebellum and hippocampus in cell survival-related functions or cell activity-related functions at one hour of hypoxia (Figures 4A, B). However, after three hours of hypoxia and after one hour of re-oxygenation there were many more genes up-regulated in cerebellum compared to hippocampus for virtually every cell survival-related functions, and consistently, cell activity-related functions including gene expression regulation (Figures 4A, B).

### Hypoxia responsive pathways in the cerebellum

The signaling pathways for those genes up-regulated only in the cerebellum but not in the hippocampus (1241 transcripts with at least 1.2 fold change) were examined using the Ingenuity Knowledge Base (Table 3 P < 0.1). Many expected pathways were identified including HIF-mediated hypoxia signaling, VEGF signaling, the NRF2-mediated oxidative stress response pathway and others. Together with the pathway analysis in the forebrain (Table 1), the results indicate that insulin-like growth factor 1 (IGF-1), glucocorticoid receptor signaling and the vitamin D3 receptor/retinoid X receptor (VDR/RXR) signaling play important roles in both the cerebellum and hippocampus. However, each of these pathways has engaged different sets of genes in cerebellum compared to hippocampus and other brain regions. Genes in other pathways, such as the integrin signaling and protein ubiquitination pathways, were exclusively up-regulated only in cerebellum (data not shown). It is notable that inflammation-related signaling is also represented in these cerebellum specific, hypoxia-regulated genes including Fcγ receptor-mediated phagocytosis signaling, interleukin-8 (IL-8) and IL-12 signaling (Table 3).

**Table 3:** Canonical signaling pathways associated with region-specific up-regulated genes in the cerebellum following HP.

Pathway	No. of Genes	P	Genes
Hypoxia Signaling in the Cardiovascular System	10	0.0003	UBE2V2, BIRC6, JUN, UBE2G2, UBE2R2 (includes EG:54926), HIF1A, EDN1, PTEN, UBE2G1, VHL

Integrin Signaling	18	0.0011	ARF6, PIK3CA, CRKL, ITGB5, PXN, FYN, DOCK1, PTK2, RHOU, ITGA6, RHOT1, TSPAN5, RHOJ, BCAR1, BCAR3, PTEN, RHOV, ZYX

VEGF Signaling	10	0.0021	PRKCB1, EIF1AY, EIF1, FOXO1, EIF2S2, PIK3CA, PXN, HIF1A, FOXO3, PTK2

Fc gammar Receptor-mediated Phagocytosis in Macrophages and	9	0.0195	PRKCB1, ARF6, PRKCD, PXN, FYN, VAMP3, DGKB, DOCK1, PTEN

Protein Ubiquitination Pathway	14	0.0204	UBE2V2, PSMC6, FBXW7, UBE2R2 (includes EG:54926), CUL2, PSMA1, VHL, USP7, BIRC6, UBE2G2, TRAF6, USP53, USP2, UBE2G1

IL-8 Signaling	13	0.0269	PRKCB1, PRKCD, GNG4, PIK3CA, GNB2L1, PTK2, ARAF, RHOU, RHOT1, TRAF6, RHOJ, RHOV, GNA13

14-3-3-mediated Signaling	12	0.0295	ARAF, PRPF4B, PRKCB1, JUN, DYRK1A, CDK7, PRKCD, FOXO1, PIK3CA, PLCD1, TUBA4A, CSNK1A1

Selenoamino Acid Metabolism	4	0.0302	MAT2A, GGT7, SEPHS2, MARS2

IGF-1 Signaling	8	0.0339	IGFBP1, JUN, FOXO1, PIK3CA, CTGF, PXN, FOXO3, PTK2

Estrogen Receptor Signaling	9	0.0427	TAF11, IGFBP1, CDK7, POLR2D, TAF9, DDX5, TAF5L, PPARGC1A, NCOA2

VDR/RXR Activation	7	0.0457	PRKCB1, IGFBP1, PRKCD, FOXO1, CEBPB, MXD1, NCOA2

NRF2-mediated Oxidative Stress Response	12	0.0525	PRKCB1, JUN, PRKCD, HSPB8, SOD1, PIK3CA, HERPUD1, CUL3, DNAJC5, FKBP5, DNAJC11, MAF

Tight Junction Signaling	11	0.0617	CTNNA1, EPB41, PPP2CA, CPSF6, JUN, CSTF1, CNKSR3, CSTF3, MYH7B (includes EG:668940), VAPA, PTEN

IL-12 Signaling and Production in Macrophages	8	0.0631	JMJD6, PRKCB1, JUN, PRKCD, PIK3CA, TRAF6, CEBPB, MAF

Glucocorticoid Receptor Signaling	15	0.0933	TAF11, AGT, CDK7, SMARCD2, PIK3CA, CEBPB, FKBP5, TAF9, TAF5L, NCOA2, JUN, NR3C2, POLR2D, TRAF6, GTF2A2

PTEN Signaling	7	0.0955	FOXO1, PIK3CA, CNKSR3, BCAR1, FOXO3, PTEN, PTK2

A total of 1,241 transcripts that were up-regulated at least 1.2-fold in the cerebellum, but not in the hippocampus, were included in the analysis. The Fisher's exact test was used to determine the significance of each signaling pathway; significance was defined as a P value less than 0.1.

### Hypoxia regulated, cerebellum specific HNF4A target genes

**Figure 5:**
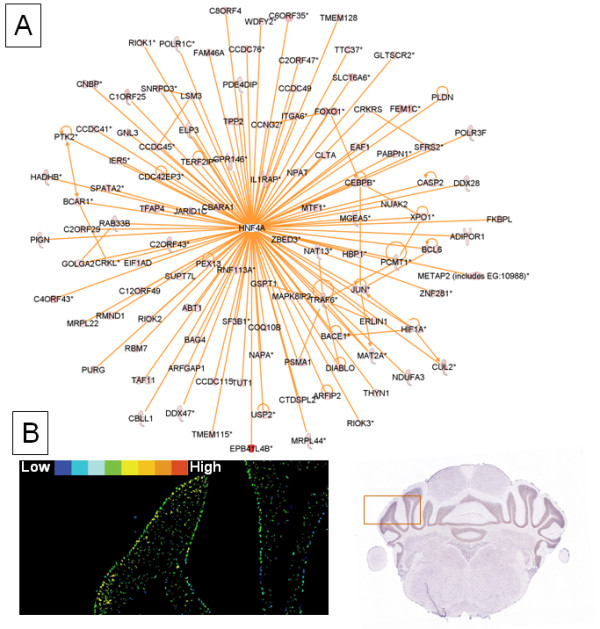
**Central role of transcription factor HNF4A in the region-specific response of cerebellum to hypoxia**. **(A)**. Network diagram showing the interactions between HNF4A and its numerous target genes that were up-regulated by hypoxia at least 1.2-fold in the cerebellum, but not in the hippocampus. The orange lines indicate interactions between molecules. **(B)**. Baseline expression pattern of HNF4A at the cellular level in the cerebellum of 8-week old male C57BL/6J mice using *in situ *hybridization(ISH) (Image ID 1746, Specimen ID 2397, Position 1875 from Allen Mouse Brain Atlas, Allen Institute for Brain Science, Seattle, WA, http://mouse.brain-map.org, accessed in April 2009). The left panel is the high power view of HNF4A expression in cerebellum cortex (the framed area in the right panel). The expression level is color coded. The right panel is the original Allen Mouse Brain Atlas image of the whole cerebellum (coronal section).

According to the above pathway analysis, transcription factors, including glucocorticoid receptor, estrogen receptor and VDR/RXR, along with HIF played significant roles in mediating the cerebellum region-unique gene expression responses (Table 3), though this did not explain the entire cerebellum-specific response. To further address this question, the 1,241 transcripts up-regulated only in cerebellum with at least 1.2 fold change were subjected to network analysis in the Ingenuity Knowledge Database. Remarkably, the transcription factor hepatic nuclear receptor 4A (HNF4A) was the hub molecule for a large network that exhibited protein-DNA interactions with the genes corresponding to 186 transcripts up-regulated only in cerebellum but not in hippocampus (Figure 5). The genes represented by the 186 transcripts are all verified HNF4A target genes using ChIP-on-chip (chromatin immunoprecipitation combined with microarray chip) [20]. The presence of such a large number of HNF4A target genes in the given list of cerebellum unique genes was statistically significant (P = 0.0001, Chi-square test). In contrast, only 23 HNF4A potential target genes were up-regulated by hypoxia in both cerebellum and hippocampus.

As the whole cerebellum was used in our current study, we sought evidence for the cellular localization of HNF4A using the Allen Mouse Brain Atlas (8-week old male C57BL/6J mice; *in situ *hybridization of ~20,000 genes; Allen Institute for Brain Science, Seattle, WA, http://mouse.brain-map.org). A picture from this atlas (Figure 5B) shows HNF4A mRNA in most if not all Purkinje cells of the cerebellum and in scattered cells in the granule cell layers (Figure 5B). HNF4A expression is almost absent in the molecular layers and in the deep nuclei of cerebellum (not shown). This restricted expression in a minority of the cells in cerebellum may explain the failure to detect HNF4A mRNA in whole cerebellum using RT-PCR (Mouse Genome Database, The Jackson Laboratory, Bar Harbor, Maine, http://www.informatics.jax.org) or on microarrays [21] or in our current study. HNF4A mRNA in situ hybridization has yet to be done in hypoxic cerebellum where cellular localization might change considerably.

Since there has not been any study of potential HNF4A target genes specifically in the cerebellum, a promoter site analysis was therefore performed in Genomatix software to identify additional potential target genes of HNF4A. The results show that 54% (642 genes) of the hypoxia-regulated genes seen only in cerebellum have HNF4A binding sites in their promoter regions. A total of 381 of the 642 genes have two or more binding sites for HNF4A. More interestingly, the integrin signaling pathway, which was exclusively up-regulated in cerebellum but not in hippocampus, was the most significant signaling pathway regulated by the 642 potential HNF4A target genes in the cerebellum.

## Discussion

The results demonstrate both time- and region-dependent gene expression responses to hypoxia. Of the 2324 hypoxia-regulated transcripts, most are specifically regulated in just a few brain regions. Some of them are under the control of similar transcription factors across regions like HIF-1α, GR and VDR/RXR, while others are not. A unique finding is that a large number of cerebellum-specific hypoxia responsive genes appear to be uniquely regulated by the HNF4A transcription factor. The finding of more up-regulated than down-regulated genes in cerebellum and other hindbrain structures compared to more down-regulated genes in forebrain, points to possible sacrifice of forebrain cognitive functions for support of life-preserving hindbrain functions during periods of marked hypoxic stress.

Predominant down-regulation of genes over up-regulation in forebrain following hypoxia could conserve energy but compromise specific forebrain functions. However, there are still many up-regulated genes in forebrain following hypoxia, and these are more involved in regulation of cell survival and cell growth than the down-regulated genes. This selective up regulation of cell survival genes, moreover, was a common feature for all the other investigated brain regions. Specific pathways included those for HIF signaling, glucocorticoid signaling, and P53 signaling, PI3K/AKT and ERK/MAPK signaling. Though the degree of hypoxia used in this study does not produce cell death [3,22,23], it may produce diffuse single strand DNA breaks that require repair [24,25]. This may account for the up-regulation of p53, PI3K/AKT and other pathways involved in DNA repair [26]

Among the hypoxia regulated genes, many are known HIF target genes [5,27,28]. Though HIF-1α plays a pivotal role in hypoxia sensing and signal transduction, its role in mediating hypoxia induced preconditioning effect is controversial, with several studies suggesting that HIF-1α might promote ischemic injury [29-32]. Microarray studies with brain specific HIF-1α knock out mouse even show that HIF-1α is dispensable for the hypoxia response[31]. If HIF related responses do not account for hypoxia preconditioning, then there must be other transcription factors and genes. Indeed, over half of the hypoxia regulated genes common to all brain regions lack HIF binding sites in their promoters but have sites for other hypoxia responsive transcription factors.

The glucocorticoid transcription regulation pathways are not commonly thought of as being associated with hypoxia and other types of preconditioning. However, these pathways might be considered since our promoter analyses show that genes with GRE (Glucocorticoid Responsive Elements) in their promoters are over-represented in those induced by one hour of hypoxia(Table 2), which is sufficient to produce hypoxia preconditioning [8]. Glucocorticoids are systemic stress hormones and can activate the glucocorticoid receptor (GR), a transcription factor that acts on GRE promoter elements [33]. Although the role of glucocorticoids in the context of HP has not been addressed, it is known that hypoxia (8% oxygen) increases glucocorticoids in the blood within 30-60 minutes [34-36]. Glucocorticoids can augment the expression of HIF1α-dependent genes via a direct interaction with HIF-1α [37]. Administration of synthetic glucocorticoids can decrease high altitude sickness, i.e. brain edema due to hypoxia [38]. Glucocorticoids applied 20 hours in advance can protect rat neurons from excitotoxin-induced apoptosis through PI3K/Akt-dependent phosphorylation of Cdkn1a [39]. Of note, our data show that hypoxia increased the expression of *Cdkn1a *(Fig. S2). According to our data, hypoxia induces many other glucocorticoid-inducible genes, including *Tsc22d3, Sgk1*, and *Nfkb1a*, as well as a member of the GR complex - FKBP5 (FK506 binding protein 5). Thus, glucocorticoid signaling could produce an alarm response to the hypoxia stress to augment the adaptive genomic response. Whether GR signaling plays a role in preconditioning has yet to be tested.

There is probably not a single pro-survival molecule or one pro-survival signaling pathway that accounts for hypoxia preconditioning. The redundant activation of multiple pro-survival mechanisms has been observed in many models, including hypoxia and ischemic preconditioning models [9,17,40]. Cellular protection may occur at many levels - and thus multiple pathways are needed to protect the cells as shown in Table 1.

The hypoxia preconditioning response appears to have little in common with an injury response. For example, very few transcripts were regulated by both hypoxia and ischemic stroke in the cerebral cortex (Additional file 2 table S5). A similar result has been found between an ischemic preconditioning and an ischemic stroke-induced gene expression response [17]. This result indicates that the protective effect induced by hypoxia is not achieved through a simple rehearsal of the ischemia event, but through a reprogramming of the transcriptional response [17].

Unlike the forebrain, there were many more up-regulated than down-regulated genes in cerebellum and other hindbrain structures including the pons and medulla. This could be due to the fact that brainstem and cerebellum play critical roles in maintaining cardiac and respiratory functions during periods of severe hypoxia and that higher order cognitive functions are shut down to conserve energy stores during hypoxia[41-43]. Indeed, hypoxic mice and other mammals are lethargic and inactive, but have marked increases of respiration, heart rate and autonomic responses during hypoxia. Our findings on the regional differences serve to emphasize that some aspects of "hypoxia preconditioning" *in vivo *may not be recapitulated by hypoxia or ischemia preconditioning *in vitro*.

The large number of up-regulated genes in cerebellum following hypoxia could suggest an important central role for cerebellum in the brain's preconditioning response. As noted above, cerebellum may play a role in coordinating respiratory, circulatory and other autonomic responses to hypoxia[41,42]. Though possible, it is not clear why there would not be similar numbers of up-regulated genes in the pons and medulla where these functions are primarily controlled. An alternative role for cerebellum in hypoxia preconditioning might occur through "central neurogenic neuroprotection," which is produced by stimulation of the cerebellum fastigial nucleus (FN) [44]. Reis and coworkers found that one hour of electrical stimulation of the FN prior to injury protected the rat brain from subsequent focal ischemia 1-3 days later [45,46]. The time course of this central neurogenic neuroprotection is similar to that of hypoxia preconditioning. The mechanism of neuroprotection is unknown, though FN stimulation pretreatment can decrease inflammation following ischemia and can modify the intrinsic sensitivity of forebrain neurons to apoptotic stimuli [47,48]. The many up-regulated genes in cerebellum produced by hypoxia imply that the cerebellum was indeed "stimulated." It is tempting to speculate that changes of gene expression in cerebellum during the hypoxia may be responsible, at least in part, for the hypoxia-induced neuroprotective effect through the "central neurogenic protection" mechanism. Indeed, an excitotoxic lesion of FN neurons prior to preconditioning ischemia blocks the neuroprotective effect of ischemic preconditioning against global ischemia in the rat [49]. Even though the cerebellar FN-mediated neuroprotection effect has been well documented, the neuronal circuitry from cerebellum to forebrain responsible for the preconditioning still has not been identified.

Although the cerebellum exhibits a different response to hypoxia compared to the hippocampus, the response in the cerebellum is still mediated to an extent by well-known hypoxia responsive signaling pathways. Besides HIF-1α, a series of nuclear receptor transcription factors appear to have important roles in the cerebellum-specific up regulation response, including GR, NRF2 (NF-E2-related factor 2), VDR/RXR, and estrogen receptor. One of the major findings of this study, however, is that over half of the 1241 hypoxia-regulated genes unique to cerebellum have binding sites in their promoters for HNF4A, another member of the nuclear receptor family.

As a tissue-specific transcription factor, hepatocyte nuclear factor 4A (HNF4A) was originally identified as a key transcription factor responsible for expression of hepatic-specific genes and is involved in tissue-specific cell differentiation and energy metabolism [20,50]. Mutations in this gene have been associated with monogenic autosomal dominant non-insulin-dependent diabetes mellitus type I, which stresses the important role of HNF4A in energy metabolism [51]. In addition, conditional HNF4A-knockout mice have down-regulation of a series of cell adhesion and junction molecules and severe failure of epithelial transformation [52], which is consistent with the potential HNF4A target genes regulated in the hypoxic cerebellum.

HNF4A is also known to be involved in the hypoxia response. HNF4A can interact directly with both HIF-1α and HIF-1β and is required for erythropoietin (EPO) transcription during hypoxia through interaction with HNF4A binding element in the EPO enhancer in the liver and kidney [53]. Mutation of the HNF4A binding site in the EPO promoter abolishes hypoxic induction of EPO [54]. According to our data, HNF4A appears to activate transcription for a large number of cerebellum-specific hypoxia responsive genes. Though a role for HNF4A in the hypoxia response in brain has yet to be proven, the current findings suggest that it plays a major role in the transcriptional response to hypoxia in the mammalian cerebellum.

## Conclusions

To the best of our knowledge, our data uncovered so far the most complete HP-induced gene expression responses in the adult mouse brain. Our results confirm that HP elicits both time- and region-dependent transcriptional responses that are required for maturation of the delayed protection effect of HP. Both region-independent and region-dependent expression changes were observed. Developmentally closely related regions share more commonality than remotely related regions: roughly, the response in the forebrain regions is distinctive from that in the hindbrain regions. At the same time, from the whole brain view the region-dependent response in vivo appears to be a well coordinated one under limited energy sources, with hindbrain function being well supported somewhat at the expenses of the forebrain function. Nonetheless, selective up-regulation of cell survival related genes appears to be a common feature for all the brain regions including the forebrain. A large proportion of HP-regulated genes themselves are gene expression regulators. The data analysis suggested the complexity of such underlying transcriptional regulation mechanism: both universal transcriptional regulation mechanism, such as HIF and GR, and region-specific transcription factors, such as HNF4A, have been responsible for the region-specific gene expression changes which is related to cell survival. It is likely that a cascade of signaling pathways rather than any single pathway has mediated the HP response and protection mechanism. Our data also revealed novel transcriptional regulation mechanisms that may have been underappreciated in HP neuroprotection mechanism, and indicated the potentially important role of cerebellum and other hindbrain structures in HP. The exact mechanism and function of the region-dependent responses to HP remain to be fully elucidated.

## Methods

### Animals

All studies were reviewed and approved by IACUC (Institutional Animal Care and Use) committee of the University of California at Davis. All mice had food and water available ad libitum on a 12 hour light/dark cycle, and were acclimated to the animal room for at least one week before the experiment.

### Hypoxia and Tissue Samples

C57BL/6 male mice, ages 8-9 weeks, were exposed to 8% O_2 _and 92% N_2 _for 3 hours in hypoxia chambers (BioSpherix, NY, USA) and then returned to room air in their home cages for 24 hours. Sham-treated control mice were also placed in the same chambers but with room air for 3h, and then returned to their home cages for 24h. During the experiments mouse brains were removed and dissected in a cold room (4°C) at the following time points: immediately after 1 hr of hypoxia (H1), immediately after 3 hr of hypoxia (H3), and after 1 (R1), 3 (R3), 6 (R6), 12 (R12), and 24 (R24) hours of re-oxygenation (R). Total RNA was purified from the following brain regions: cerebral cortex, hippocampus, striatum, thalamus, midbrain, cerebellum and pons/medulla. Three mice were studied for each hypoxia time point and sham condition, for a total of 24 mice studied and 168 samples (microarrays).

### RNA and microarray

Brain samples were homogenized in 1 ml of TRIZOL Reagent (Invitrogen Corporation, Carlsbad, California). RNA purification was carried out according to the manufacturer's recommendations (Protocol 18057N, Invitrogen Corporation, Carlsbad, CA). The RNA pellet was cleaned using a RNAeasy mini kit (Qiagen, Valencia, CA). RNA purity and integrity were assessed using a Nanodrop spectrophotometer (NanoDrop Technologies, Wilmington, DE) and an Agilent Bioanalyzer (Agilent Technologies Inc., Palo Alto, CA). Samples (5 μg of total RNA) were processed on whole genome "GeneChip® Mouse Expression 430 2.0" arrays according to the Affymetrix technical manual (Affymetrix GeneChip® Expression Analysis Manual 701023, Rev. 4, Affymetrix). Samples had to have an A260/A280 absorbance ratio greater than 1.9 and a 28S/18S rRNA ratio greater than 1.5. The raw data are available through NCBI Gene Expression Omnibus (GEO) with series accession number [GEO: GSE19709].

### Microarray Data Analysis

Raw signals were transformed into .CEL files in GCOS software (Affymetrix, Santa Clara, CA). Probe data were generated using the Robust Multi-chip Average with GC-content Background Correction (GCRMA, http://www.bioconductor.org) in Genespring 7 software (Silicon Genetics, Redwood City, CA). This involves background correction, quantile normalization, and summarization of the probe-set values into gene-level expression measurements. The expression data for each brain region of hypoxia treated mice were then normalized to the averaged values for each brain region from the sham treated mice. Differentially regulated genes were determined using a one-way ANOVA analysis and a Benjamini Hochberg False Discovery Rate (FDR) (<0.05) method for multiple comparison corrections [55], followed by a Student's post hoc test.

We performed a separate analysis that was designed to minimize the number of false negative genes. Those genes whose expression changed little under the different experimental conditions were filtered out. Specifically, only those genes whose expression changed at least 1.3-fold from the baseline value in at least two of the hypoxia-treated samples were selected for downstream analysis. This filter helped maximize the number of genes. This approach removed approximately 60% of probe sets prior to further analysis. The remaining genes were then subjected to a one-way ANOVA analysis. The resulting gene lists not only contained a significant number of genes that overlapped with the one generated by the aforementioned analysis with no pre-filtering, but it also contained a significant number of genes missed in the initial analysis without the pre-filtering step. These additional positive genes were then combined with the genes derived from the initial analysis without pre-filtering. Less stringent 1.2 fold change gene lists were used for functional analyses and more stringent 1.5-fold change gene lists were used to analyze transcription factor binding sites.

For example, in the cerebral cortex, the analysis of HP samples using pre-filtering indicated that the expression of 831 genes changed during the time course; this set of genes included 94.4% of the 531 genes generated by the same analysis without pre-filtering. The additional 339 genes in the pre-filtering analysis were added to the initial 531 genes, resulting in the final list of 860 genes. However, if a 1.5 fold change cut off was used, a total of 503 genes were identified as HP regulated genes in the cerebral cortex.

### Functional Analysis

Genes that met statistical criteria were analyzed using Ingenuity Pathways Analysis (IPA 6.5, Ingenuity® Systems, http://www.ingenuity.com) to explore molecular functions, signaling pathways and interaction networks. These analyses identified the most statistically significant biological functions or canonical pathways in the data set. Fischer's exact test was used to calculate a p-value describing the probability that a given biological function or canonical pathway was assigned to that data set due to chance alone. An interaction network is a graphical representation of the molecular relationships between genes and gene products. Gene products are represented as nodes, and the biological relationship between two nodes is represented as a line that is supported by at least one reference from the literature.

The in situ hybridization data for HNF4A mRNA expression in cerebellum were obtained from the Allen Brain Atlas, a genome-wide image database of gene expression in the brain of 8 week old C57BL/6J mice (http://mouse.brain-map.org). Non-radioactive digoxigenin-labeled HNF4A riboprobes were synthesized and hybridized to HNF4A mRNA in brain tissue sections. Detailed methods can be found in the reference for the Allen Brain Atlas [56].

### Promoter Prediction and Analysis

Transcription factor binding site (TFBS) analysis of the promoter sequences was carried out in GEMS Software (Genomatix software, www.genomatix.de). GEMS provides genome annotation, promoter sequence retrieval, a search engine for transcription factor binding sites, and a transcription factor knowledge base. Promoter sequences were extracted from 500 bp upstream and 100 bp downstream of the transcription start site of genes of interest. Although the exact length of a promoter can only be defined experimentally, a commonly accepted definition of proximal promoter region for transcription factor binding is typically 250-500 bp located directly upstream of the site of initiation of transcription in eukaryotes [57]. It is a common practice for an initial in silico analysis to refrain from extending the length beyond the proximal promoter region. This will help to minimize false positive findings for computation prediction. Genomatix develops optimized algorithm for promoter modeling and predicting based on the region between 500 bp upstream and 100 bp downstream of the transcription start site. MatInspector within GEMS was used to scan transcription factor matrix matches in input sequences based on position weight matrices. The weight matrix for a specific transcription factor binding site reflects the differences in the degree of conservation of a specific nucleotide at a specific position. In order to reduce false positives, the resulting matrix similarity score for a transcription factor was compared to the optimized threshold value which is individualized for individual transcription factor binding site matrix [58]. A p value was then determined as the probability to obtain an equal or greater number of sequences with a match in a randomly drawn sequence set of the same size as the input sequence set. The computational methods used in Genomatix have been successfully applied to identify proven functional transcription factor binding sites [59,60].

## Abbreviations

Cdkn1a: cyclin-dependent kinase inhibitor 1A; EPO: Erythropoietin; FKBP5: FK506 binding protein 5; FN: cerebellar fastigial nucleus; GR: glucocorticoid receptor; GRE: Glucocorticoid Responsive Elements; HIF: Hypoxia-inducible transcription factor; HNF4A: hepatic nuclear receptor 4A; HP: hypoxia preconditioning; IGF: Insulin-like growth factor; klf4: Krueppel-like factor 4; Nfkb1a: nuclear factor of kappa light polypeptide gene enhancer in B-cells; NRF2: NF-E2-related factor 2. PI3K/AKT: Phosphatidylinositol-3-kinase/Protein kinase B; Sgk1: serum/glucocorticoid regulated kinase 1(Previous name: serum/glucocorticoid regulated kinase); Tsc22d3: TSC22 domain family, member 3(Previous name: Glucocorticoid-induced leucine zipper protein); VDR/RXR: Vitamin D Receptor/Retinoic Acid X Receptor;

## Authors' contributions

HX and FRS conceived of the study. HX, AL performed the experiments. All the authors participated in its design and coordination. HX analyzed the data and drafted the manuscript. All authors read and approved the final manuscript.

## Additional data files

The following additional data files are available with the online version of this paper: Additional file 1 contains Figure S1 to S5; Additional file 2 contains Table S1 to S5.

## Supplementary Material

Additional file 1 **Figure S1. Classification overview of the hypoxia-regulated genes in the brain**. (A). Sub-cellular localization of proteins encoded by these genes. (B). Molecular type classifications of the gene products. Figure S2. Time course of expression of selected HIF-1 alpha target genes in all brain regions studied. Diagram of the expression activities of *Cdkn1a *(Cyclin-dependent kinase inhibitor 1A (P21)) and *Ddit4 *(DNA damage-inducible transcript) over the time course after HP treatment in each brain region. Figure S3. Cluster analysis diagram of the expression profiles from each forebrain region. Expression heatmap and cluster tree diagram showing the common expression pattern of the 300 transcripts over the time course after HP treatment in all three forebrain regions. Figure S4. Interactive signaling network associated with the genes up-regulated by HP in all the forebrain regions. Diagram of the interplay among various signaling pathways during the responses to HP treatment. Figure S5. Expression of krueppel-like binding factor 4 (*Klf4*) in the forebrain regions after HP. Shows the expression changes of *Klf4 *genes, represented by two probes, in each forebrain region over the time course after preconditioning hypoxia. Click here for file

Additional file 2 **Table S1. Verified HIF-1 alpha target genes regulated by HP**. List of verified 17 HIF-1 alpha target genes which are regulated by HP in at least one of the brain regions investigated. Table S2. Computationally predicted HIF-1alpha target genes whose expression was regulated by HP. List of 38 predicted HIF-1alpha target genes which are regulated by HP in all brain regions. Table S3. Molecular functions significantly associated with the top three networks formed by HP-regulated genes in each forebrain region. The top networks were ranked by their significance scores determined by network analysis in Ingenuity database. Table S4. Transcripts commonly up-regulated in all the three forebrain regions. List of 87 transcripts commonly up-regulated in cerebral cortex, hippocampus and striatum. The time points at which the transcript showed statistically significant expression change are provided together with corresponding fold change value. Table S5. Common genes regulated by both hypoxia preconditioning and acute ischemia in the brain. List of genes commonly regulated in both mice HP model and rodent (mice or rat) MCAO model. Click here for file
