# Supplementary material for: Regional genome transcriptional response of adult mouse brain to hypoxia
Source: BMC Genomics. 2011 Oct 11;12:499. doi: 10.1186/1471-2164-12-499 (PMC3218040; doi:10.1186/1471-2164-12-499)
Supplement: Additional file 1 — Figure S1. Classification overview of the hypoxia-regulated genes in the brain. (A). Sub-cellular localization of proteins encoded by these genes. (B). Molecular type classifications of the gene products. Figure S2. Time course of expression of selected HIF-1 alpha target genes in all brain regions studied. Diagram of the expression activities of Cdkn1a (Cyclin-dependent kinase inhibitor 1A (P21)) and Ddit4 (DNA damage-inducible transcript) over the time course after HP treatment in each brain region. Figure S3. Cluster analysis diagram of the expression profiles from each forebrain region. Expression heatmap and cluster tree diagram showing the common expression pattern of the 300 transcripts over the time course after HP treatment in all three forebrain regions. Figure S4. Interactive signaling network associated with the genes up-regulated by HP in all the forebrain regions. Diagram of the interplay among various signaling pathways during the responses to HP treatment. Figure S5. Expression of krueppel-like binding factor 4 (Klf4) in the forebrain regions after HP. Shows the expression changes of Klf4 genes, represented by two probes, in each forebrain region over the time course after preconditioning hypoxia. [file 1471-2164-12-499-S1.PDF]

Additional File 1 Figure Legends

Additional file 1, figure S1.

Classification overview of the hypoxia–regulated genes in the brain. (A) Sub-cellular localization of proteins encoded by genes whose expression is altered by hypoxia. The pie diagram was constructed based on annotations from the Ingenuity Knowledge Base and the Affymetrix Netaffy Tool. The size of the slice representing each cellular compartment within the pie indicates the relative percentage of gene products located in that compartment. A total of 1389 transcripts were classified whose protein products have subcellular localizations described in the database. The expression of these transcripts is altered at least 1.5-fold by HP.

(B) Overview of the types of the gene products regulated by HP in at least one brain region based on the annotations in the Ingenuity Knowledge Base. A total of 853 transcripts were classified whose expression is changed at least 1.5-fold by HP and for which molecular type annotations exist in the database. The x-axis indicates the various molecular type classifications, and the y-axis gives the total number of transcripts associated with each type of molecule.

Additional file 1, figure S2.

Time course of expression of selected HIF-1 alpha target genes in all brain regions studied. Cdkn1a: Cyclin-dependent kinase inhibitor 1A (P21). Ddit4: DNA damage-inducible transcript. The y-axis is the expression value (normalized and log-transformed). The x-axis shows the time course after HP in the following order: C, control; H1, immediately after 1 hr of hypoxia; H3, immediately after 3 hr of hypoxia; R1, 1 hr after reoxygenation; R3, 3 hr after reoxygenation; R6, 6 hr after reoxygenation; R12, 12 hr after reoxygenation; R24, 24 hr after reoxygenation.

Regions: cerebral cortex (Cortex), hippocampus (Hippo.), striatum (Stri.), thalamus (Thal.), midbrain (Mid.), pons and medulla (Pon.), cerebellum (Cere.).

Additional file 1, figure S3.

Cluster analysis diagram of the expression profiles from each forebrain region.

This analysis is based on the 300 transcripts regulated in all three forebrain regions. Each column represents individual conditions having the characteristics labeled along the x-axis: The three forebrain brain regions are distinguished from each other using colored squares along the x-axis (red, cerebral cortex; blue, hippocampus; purple, striatum); the time point is indicated below the x-axis. Each row represents an individual transcript. The average expression value of each transcript in each condition is color-coded, with red high and green low, as shown in the color bar to the right of the figure. Conditions with similar expression profiles were indicated by the closer distances of the corresponding branches in the tree structure at the top of the figure. The same time points from different regions cluster together, suggesting that different forebrain regions share similar expression profiles along the time course. Transcripts with similar expression activities across all conditions clustered together, as shown in the tree diagram on the y-axis. The top cluster of rows of transcripts contains those up-regulated by HP in the forebrain regions.

Additional file 1, figure S4.

Interactive signaling network associated with the genes up-regulated by HP in all the forebrain regions. For this network analysis, 30 genes from 87 transcripts that were up-regulated in all the forebrain regions were selected based on their known association with cell

death/survival or growth regulation according to published studies. The light green lines indicate varieties of interactions between the connected molecules, such as protein-protein binding, phosphorylation, and activation. Selective molecules are color-coded according to the corresponding signaling pathways. For example, red represents glucocorticoid receptor signaling; dark red, HIF-1 alpha signaling pathways; orange, the P53 pathway; blue, the ERK/MAPK pathway; pink, the insulin/IGF-1 pathway.

Additional file 1, figure S5.

Expression of krueppel-like binding factor 4 (Klf4) in the forebrain regions after HP.

The x-axis shows the time course in each forebrain region after preconditioning hypoxia in the following order: C, control; H1, immediately after 1 hr of hypoxia; H3, immediately after 3 hr of hypoxia; R1, 1 hr after reoxygenation; R3, 3 hr after reoxygenation; R6, 6 hr after reoxygenation; R12, 12 hr after reoxygenation; R24, 24 hr after reoxygenation. This gene was measured by two probe sets on the Affymetrix MU430 2.0 array, and both results correlated with each other. The y-axis is the normalized gene expression value.

Fig. S1A

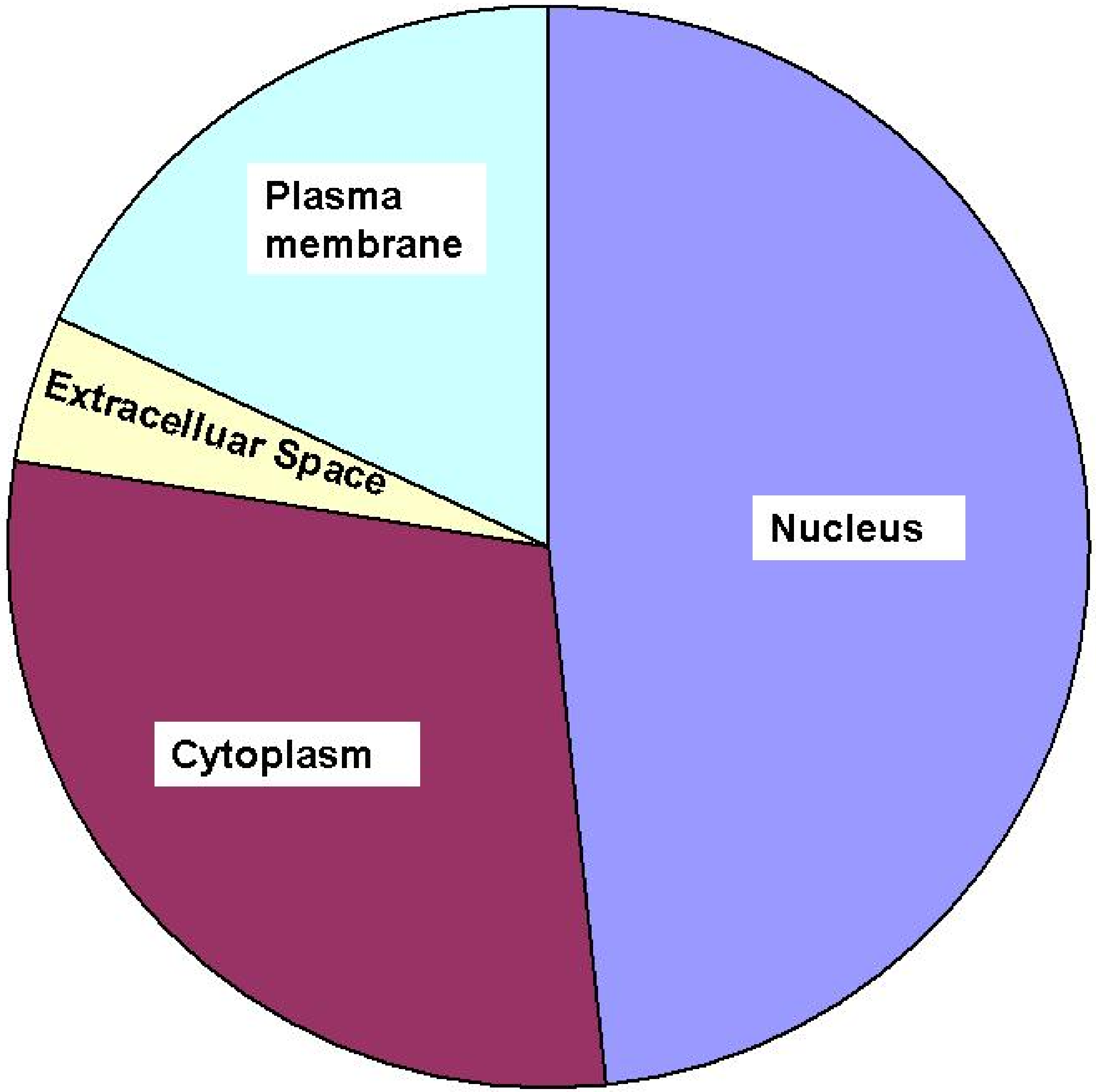

Fig. S1B

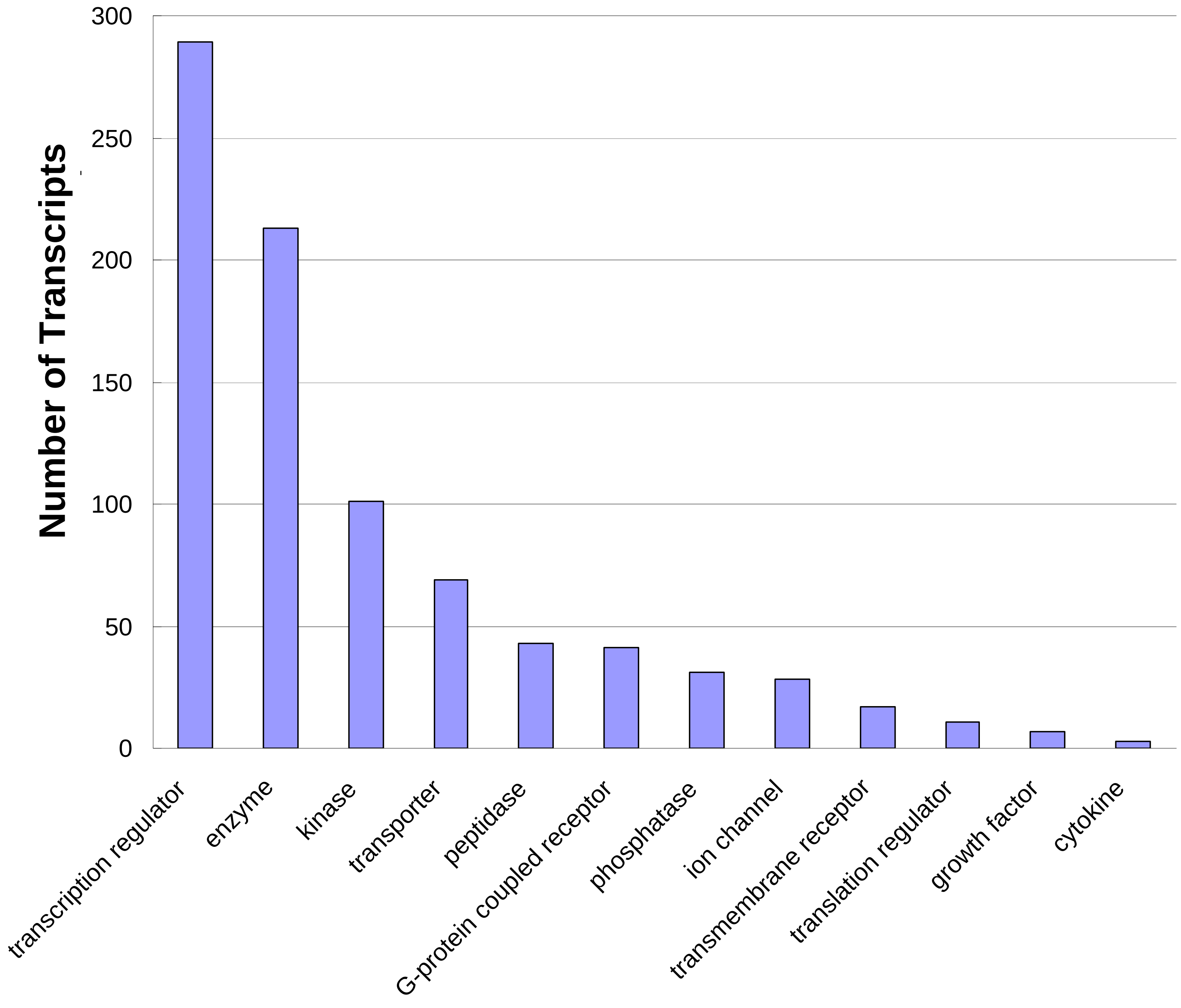
Number of Transcripts
300
250
200
150
100
50
0
transcription regulator
enzyme
kinase
transporter
peptidase
G-protein coupled receptor
phosphatase
ion channel
transmembrane receptor
translation regulator
growth factor
cytokine

Fig. S2

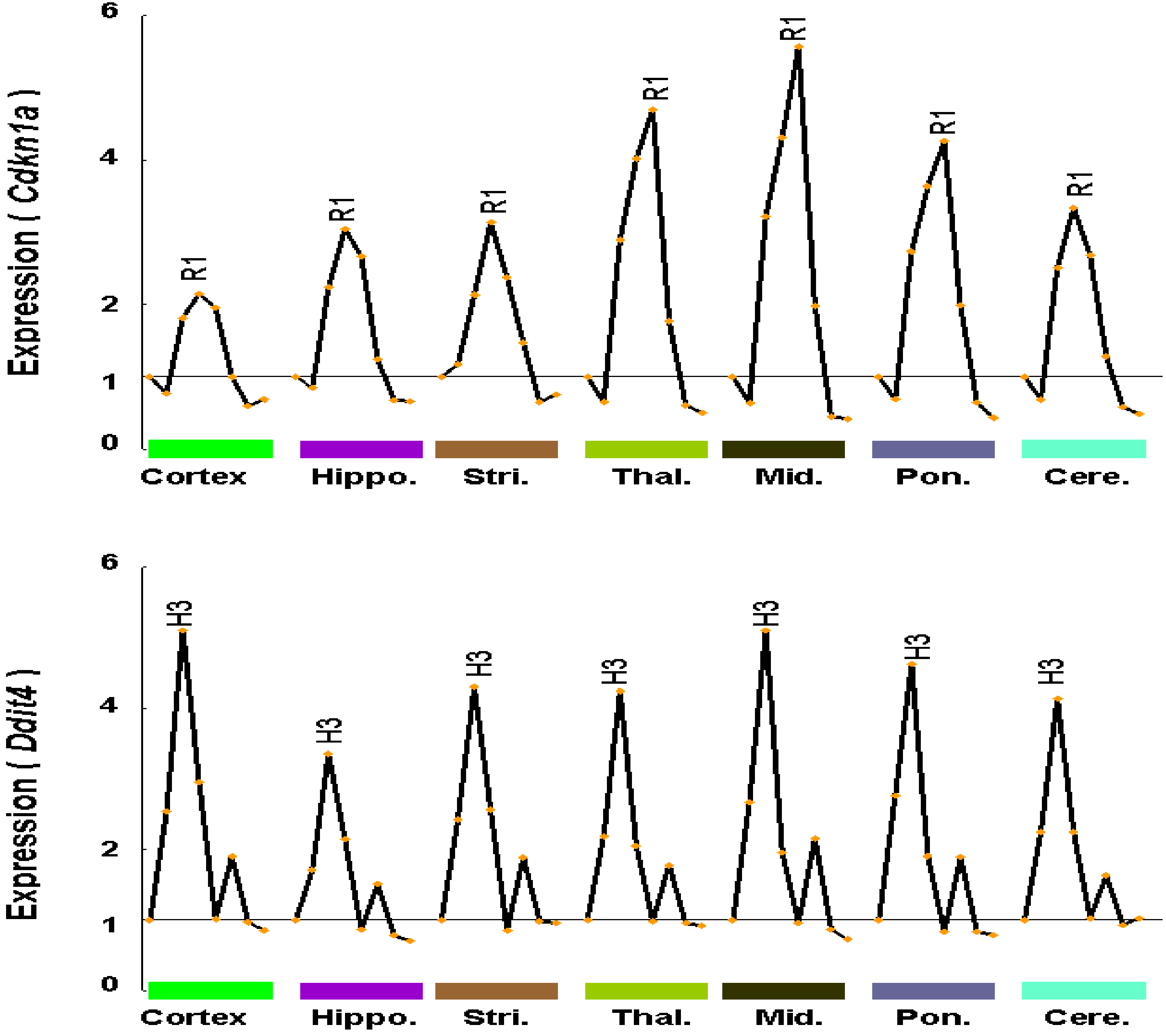

6
4
2
1
0
Expression (Cdkn1a)
R1
R1
R1
R1
R1
R1
R1
Cortex
Hippo.
Stri.
Thal.
Mid.
Pon.
Cere.
6
4
2
1
0
Expression (Ddit4)
H3
H3
H3
H3
H3
H3
H3
Cortex
Hippo.
Stri.
Thal.
Mid.
Pon.
Cere.

Fig. S3

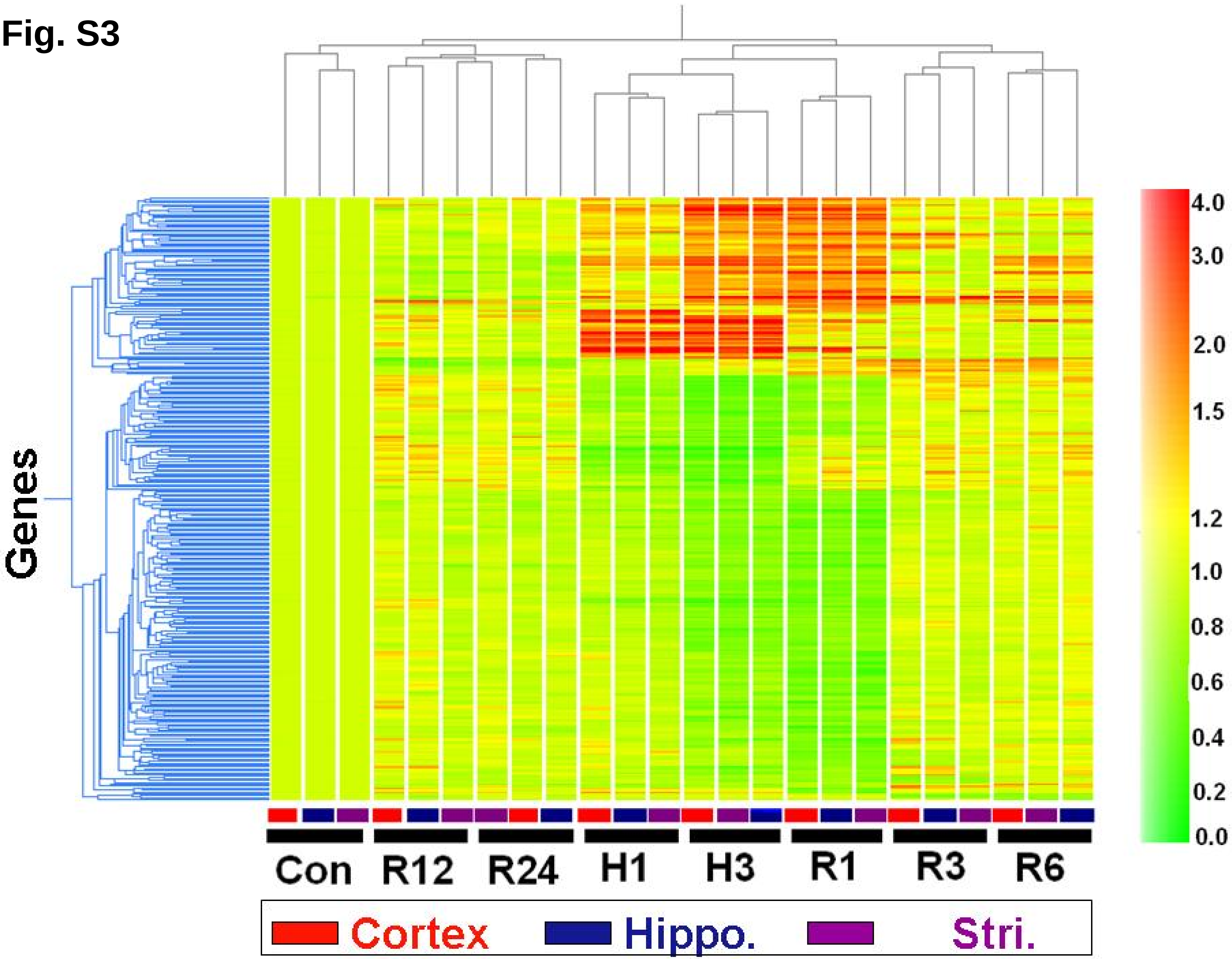

Genes
4.0
3.0
2.0
1.5
1.2
1.0
0.8
0.6
0.4
0.2
0.0
Con
R12
R24
H1
H3
R1
R3
R6
Cortex
Hippo.
Stri.

Fig. S4

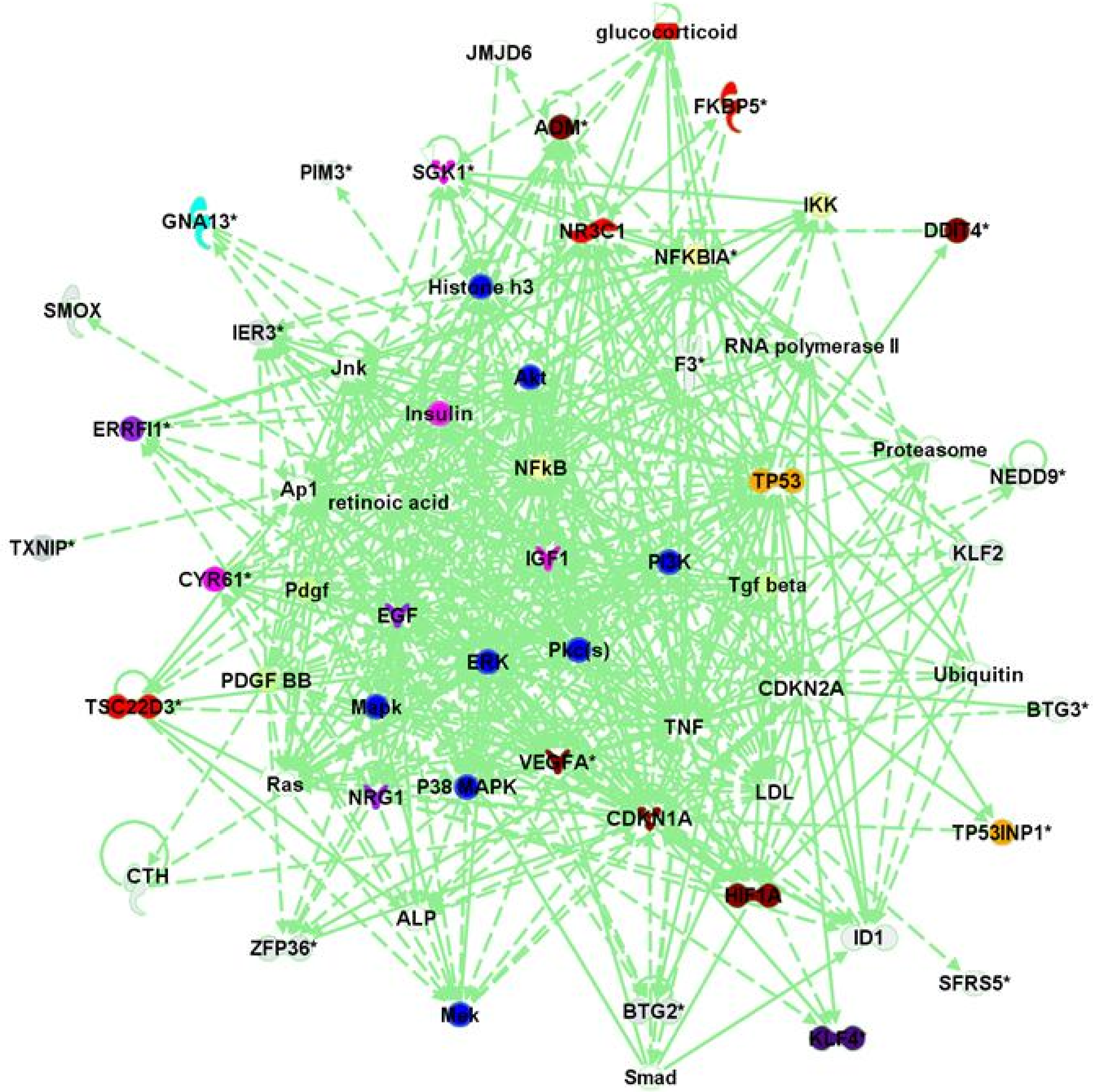

© 2000-2008 Ingenuity Systems, Inc. All rights reserved.

Fig. S5

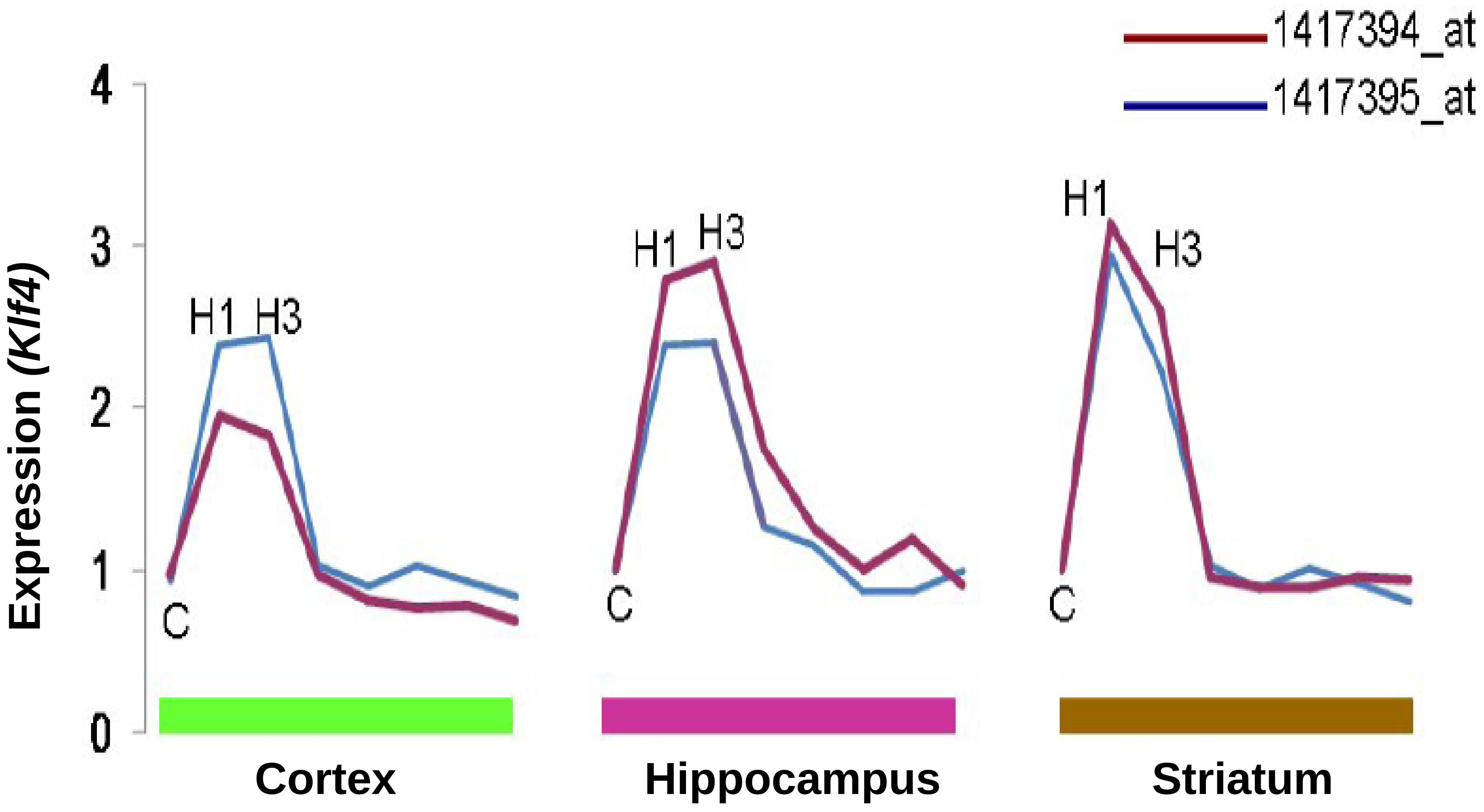

1417394_at
1417395_at
4
3
2
1
0
Expression (*Klf4*)
H1 H3
C
H1 H3
C
H1
H3
C
Cortex
Hippocampus
Striatum
