# Supplementary material for: Regional genome transcriptional response of adult mouse brain to hypoxia
Source: BMC Genomics. 2011 Oct 11;12:499. doi: 10.1186/1471-2164-12-499 (PMC3218040; doi:10.1186/1471-2164-12-499)
Supplement: Additional file 2 — Table S1. Verified HIF-1 alpha target genes regulated by HP. List of verified 17 HIF-1 alpha target genes which are regulated by HP in at least one of the brain regions investigated. Table S2. Computationally predicted HIF-1alpha target genes whose expression was regulated by HP. List of 38 predicted HIF-1alpha target genes which are regulated by HP in all brain regions. Table S3. Molecular functions significantly associated with the top three networks formed by HP-regulated genes in each forebrain region. The top networks were ranked by their significance scores determined by network analysis in Ingenuity database. Table S4. Transcripts commonly up-regulated in all the three forebrain regions. List of 87 transcripts commonly up-regulated in cerebral cortex, hippocampus and striatum. The time points at which the transcript showed statistically significant expression change are provided together with corresponding fold change value. Table S5. Common genes regulated by both hypoxia preconditioning and acute ischemia in the brain. List of genes commonly regulated in both mice HP model and rodent (mice or rat) MCAO model. [file 1471-2164-12-499-S2.PDF]

Supplementary Table S1. Seventeen verified HIF-1 alpha target genes regulated by HP.

Gene Symbol	Description	Genbank	Representative Probe ID
Adm*	**adrenomedullin**	**NM_009627**	**1416077_at**
Cdkn1a*	**cyclin-dependent kinase inhibitor 1A (P21)**	**NM_007669**	**1421679_a_at**
Ets1*	**E26 avian leukemia oncogene 1, 5' domain**	**BB151715**	**1452163_at**
Ddit4*	**DNA-damage-inducible transcript 4**	**AK017926**	**1428306_at**
Vegfa*	**vascular endothelial growth factor A**	**NM_009505**	**1420909_at**
Edn1	endothelin 1	D43775	1451924_a_at
Bhlhb2	basic helix-loop-helix domain containing, class B2	NM_011498	1418025_at
Cited2	Cbp/p300-interacting transactivator, with Glu/Asp-rich carboxy-terminal domain, 2	Y15163	1452207_at
Ctgf	connective tissue growth factor	NM_010217	1416953_at
Cxcl12	chemokine (C-X-C motif) ligand 12	NM_013655	1417574_at
Idb2	inhibitor of DNA binding 2	NM_010496	1422537_a_at
Igfbp1	insulin-like growth factor binding protein 1	NM_008341	1418918_at
Mt1	metallothionein 1	BC027262	1451612_at
Nr4a1	nuclear receptor subfamily 4, group A, member 1	NM_010444	1416505_at
Nt5e	5' nucleotidase, ecto	AV273591	1428547_at
Pfkfb3	6-phosphofructo-2-kinase/fructose-2,6-biphosphatase 3	NM_133232	1416432_at
Pmaip1	phorbol-12-myristate-13-acetate-induced protein 1	NM_021451	1418203_at

* Regulated by HP in all the brain regions investigated

Supplementary Table S2. Thirty-eight genes that are regulated by HP in all brain regions and whose promoter regions have potential HIF transcription factor family binding sites

**Gene Symbol**	**Gene Title**	**GO biological process term**	**GeneBank ID**
1190005F20Rik	RIKEN cDNA 1190005F20 gene	tRNA processing /// tRNA processing	AK004493
4930535B03Rik	RIKEN cDNA 4930535B03 gene	---	BM206427
Ap2a2	adaptor protein complex AP-2, alpha 2 subunit	protein complex assembly /// transport /// intracellular protein tra	AK009735
Apold1	apolipoprotein L domain containing 1	---	BM123813
Arrdc2	arrestin domain containing 2	---	AW542672
Arrdc3	arrestin domain containing 3	---	BG072824
Axud1	AXIN1 up-regulated 1	apoptosis	BG070296
Btg2	B-cell translocation gene 2, anti-proliferative	transcription /// regulation of transcription, DNA-dependent /// pı	NM_007570
Btg2	B-cell translocation gene 2, anti-proliferative	transcription /// regulation of transcription, DNA-dependent /// pı	NM_007570
Ccdc76	coiled-coil domain containing 76	---	BG085812
Cdkn1a	cyclin-dependent kinase inhibitor 1A (P21)	response to DNA damage stimulus /// cell cycle /// cell cycle arr	AK007630
Clk1	CDC-like kinase 1	protein amino acid phosphorylation /// peptidyl-serine phosphor	U21209
Ddit4	DNA-damage-inducible transcript 4	apoptosis /// negative regulation of signal transduction	AK017926
Eltd1	EGF, latrophilin seven transmembrane domain containing 1	signal transduction /// G-protein coupled receptor protein signal	BC017134
Errfi1	ERBB receptor feedback inhibitor 1	stress-activated protein kinase signaling pathway	NM_133753
Ets1	E26 avian leukemia oncogene 1, 5' domain	transcription /// regulation of transcription, DNA-dependent /// re	BB151715
F3	coagulation factor III	blood coagulation /// blood coagulation	BC024886
Fbxo30	F-box protein 30	ubiquitin cycle	BB706685
Galnt7	UDP-N-acetyl-alpha-D-galactosamine: polypeptide N-acetylgalactosaminyltransferase 7	protein amino acid O-linked glycosylation	AV302406
Gm129	gene model 129, (NCBI)	---	BB407125
Gpr146	G protein-coupled receptor 146	signal transduction /// G-protein coupled receptor protein signal	BQ177047
H3f3b	H3 histone, family 3B	nucleosome assembly	BM241237

Supplementary Table S2. (Cont')

Gene Symbol	Gene Title	GO biological process term	GeneBank ID
Hmgb2	high mobility group box 2	base-excision repair, DNA ligation /// base-excision repair, DNA	C85885
Hmgb2	high mobility group box 2	base-excision repair, DNA ligation /// base-excision repair, DNA	X67668
Id3	inhibitor of DNA binding 3	negative regulation of transcription from RNA polymerase II pro	NM_008321
Ier3	immediate early response 3	---	NM_133662
Jag1	jagged 1	morphogenesis of an epithelial sheet /// cell communication /// N	AV359819
Klf4	Kruppel-like factor 4 (gut)	transcription /// regulation of transcription, DNA-dependent /// n	BG069413
Klf4	Kruppel-like factor 4 (gut)	transcription /// regulation of transcription, DNA-dependent /// n	BG069413
Mt2	metallothionein 2	cellular zinc ion homeostasis /// nitric oxide mediated signal tran	AA796766
Mylip	myosin regulatory light chain interacting protein	ubiquitin cycle	BC010206
Pim3	proviral integration site 3	protein amino acid phosphorylation	BB206220
Polr3e	polymerase (RNA) III (DNA directed) polypeptide E	transcription	AV251549
Polr3e	polymerase (RNA) III (DNA directed) polypeptide E	transcription	AV251549
Rsrc2	arginine/serine-rich coiled-coil 2	---	BB036922
S3-12	plasma membrane associated protein, S3-12	---	NM_020568
Thap2	THAP domain containing, apoptosis associated protein 2	---	BG073155
Tra2a	transformer 2 alpha homolog (Drosophila)	mRNA processing /// RNA splicing	BF466228
Txnip	thioredoxin interacting protein	transcription /// regulation of transcription, DNA-dependent /// re	AF173681
Txnip	thioredoxin interacting protein	transcription /// regulation of transcription, DNA-dependent /// re	AF173681
Ube2b	ubiquitin-conjugating enzyme E2B, RAD6 homology (S. cerevisiae)	DNA repair /// ubiquitin-dependent protein catabolic process ///	AK011961
Vegfa	vascular endothelial growth factor A	angiogenesis /// angiogenesis /// angiogenesis /// angiogenesis	NM_009505
Vegfa	vascular endothelial growth factor A	angiogenesis /// angiogenesis /// angiogenesis /// angiogenesis	U50279
Zbtb20	zinc finger and BTB domain containing 20	---	BB087247

Supplementary Table S3. Functions significantly associated with the top three networks formed by HP-regulated genes in each forebrain region.

Top Three Networks	Analysis	Score	Top Functions	Molecules in Network	No. Molecules from input genes
1	Up Genes in Hippo.	49	Cell Cycle, Cell Death, Cancer	BACH2, BHLHB2, BTG2, CCNE2, CTH, Cyclin A, Cyclin D, Cyclin E, CYR61, DIO2, DUSP6, E2f, ERK, ERRFI1, Fibrin, FZD2, GAB1, HMGCS1, IER2, IER3, KLF4, MAFK, MT1E, MT1F, NR4A3, NUFIP1, Pdgf, PDGF BB, PPP1R15A (includes EG:23645), RPS6KB1, SFRS7, SIRT1, SLCO1A2, SPSB1, ZFP36	27
1	Up Genes in Cortex	43	Cancer, Cell Death, Reproductive System Disease	ADM, CDKN1A, CRKRS, FKBP4, FKBP5, GADD45B, H3F3B, HHEX, Histone h3, HMGB2, Hsp70, Hsp90, HSPA8, ID1, IER3, Ikb, IKK, IL1, KLF2, MT1F, NFkB, NFKBIA, NOC2L, Nos, Notch, PIM3, PPP1R15A (includes EG:23645), Proteasome, RNA polymerase II, SGK1, TIFA, TP53INP1, UBC, Ubiquitin, ZFP36	23
1	Up Genes in Striatum	38	Cancer, Cell Death, Cellular Growth and Proliferation	ADM, Ap1, BGN, BTG2, Calcineurin protein(s), CD3, CTH, CYR61, EDN1, EHD2, ERK, ERRFI1, F3, GADD45B, GC-GCR dimer, KLF2, MAFK, MAP2K1/2, Mek, MT1E, Nfat, Notch, P2RY6, PDGF BB, PDPN, PICALM, Pkc(s), PTPRJ, Raf, Ras, SLC2A1, SPSB1, TCR, TSC22D3, TXNIP	21
2	Up Genes in Hippo.	41	Cell Morphology, Cellular Movement, Cancer	ABCA1, ADM, ALP, AP2A2, ARL4D, CDKN1A, DMN, DTNB, EGR1, F3, Hsp27, ID1, IKK, KLF2, LDL, MGA (includes EG:23269), MMP14, MSC, NEDD9, NFkB, Notch, NR1D1, PIM3, Proteasome, RAD23B, RNF144B, RTN4, SLC37A4, Smad, SMAD1, SOX9, Tgf beta, Trypsin, Ubiquitin, VEGFA	24
2	Up Genes in Cortex	40	Protein Synthesis, Lipid Metabolism, Molecular Transport	ABCA1, Ap1, BTG2, C3AR1, CD3, CTH, CYR61, ERK, ERRFI1, F3, GC-GCR dimer, HMGCS1, JUN, KLF9, LDL, LDLR, LPIN1, N-cor, Nfat, NR2F2, PLEKHA2, PPARα-RXRα, PPARA, RASD1, Rxr, SAP30, Smad, SPSB1, T3-TR-RXR, TCR, THRSP, Thyroid hormone receptor, TOB1, TSC22D3, TXNIP	22
2	Up Genes in Striatum	37	Cancer, Cell Death, Reproductive System Disease	ADAMTS1, Akt, ATF3, ATF4, CEBPD, DDIT4, DMN, DYRK3, FKBP5, FSH, hCG, Histone h3, Hsp27, Hsp70, Hsp90, ID1, ID2, IER3, IKK, KLF9, NEDD9, NFkB, NFKBIA, PIM3, PP2A, PPP1R15A (includes EG:23645), Proteasome, SGK1, SH3RF1, Smad, SMG5, Ubiquitin, Vegf, VEGFA, ZFP36	21
3	Up Genes in Cortex	31	Cancer, Cellular Movement, Connective Tissue Disorders	Akt, ALP, BLNK, CBARA1, Cyclin E, DUSP16, E2f, EDN1, FZD2, GEM, GNA13, HTRA1, Ige, IGF1R, Jnk, KLF4, Mek, Mmp, MMP14, MT1E, NEDD9, P38 MAPK, Pak, Pdgf, PDGF BB, PLC gamma, Ras, RHOU, SFRS7, Tgf beta, TIMP3, Trypsin, Vegf, VEGFA, ZYX	18
3	Up Genes in Hippo.	28	Cell Death, Gene Expression, Cell Cycle	Ap1, BCL2L11, Calpain, Caspase, CCR5, CDC37L1, CHD8, Creb, DNAJA2, DUSP1, ERK1/2, GADD45G, GC-GCR dimer, H3F3A, H3F3B, hCG, Histone h3, Hsp70, Hsp90, IgG, IL1, IL12, Interferon alpha, Jnk, Mek, MLLT3, MRPS9, NFKBIA, NOC2L, ODC1, P38 MAPK, PGLYRP1, SGK1, STAT3, TXNIP	18

Supplementary Table S3(cont')

3	Up Genes in Striatum	28	Cell Cycle, Hematological System Development and Function, Immune and Lymphatic System Development and Function	ALP, AP2A2, BHLHB2, Calpain, CDKN1A, CTLA2A, CUX1, Cyclin A, Cyclin E, DCLK1, E2f, ELMO1, H3F3A, H3F3B, HMGB2, IFN Beta, IL1, IL12, Interferon alpha, KLF4, LDL, Mapk, MAS1, P38 MAPK, Pdgf, PI3K, Rac, Ras homolog, SLC16A6, SMARCA5, STAT, STAT3, Tgf beta, TP53INP1, ZBTB16	17

The top networks were ranked by their significance scores determined by network analysis. The expression of the up-regulated genes increased at least 1.2-fold relative to the control following HP. Abbreviations: Cerebral cortex (Cortex), Hippocampus (Hippo.).

Supplementary Table S4. Eighty-seven transcripts commonly up-regualated in all the three forebrain regions

Probe sets	Gene Symbol	Gene Title	Time Point(fold of expression change)				
1415996_at	*Txnip*	thioredoxin interacting protein	H1(1.6)	H3(3.8)	R1(2.7)		
1415997_at	*Txnip*	thioredoxin interacting protein	H1(1.7)	H3(4.9)	R1(3.2)		
1416039_x_at	*Cyr61*	cysteine rich protein 61	H1(7.6)	H3(3.7)	R1(2.4)		
1416041_at	*Sgk1*	serum/glucocorticoid regulated kinase 1	H1(1.7)	H3(2.7)	R1(2.4)		R6(1.9)
1416077_at	*Adm*	adrenomedullin	H1(2)	H3(5)			
1416101_a_at	*Hist1h1c*	histone cluster 1, H1c		H3(1.6)	R1(1.7)	R3(1.8)	
1416129_at	*Errfi1*	ERBB receptor feedback inhibitor 1		H3(1.7)	R1(1.6)		
1416250_at	*Btg2*	B-cell translocation gene 2, anti-proliferative	H1(3.1)				
1416926_at	*Trp53inp1*	transformation related protein 53 inducible nuclear protein 1			R1(1.7)	R3(1.6)	
1417394_at	*Klf4*	Kruppel-like factor 4 (gut)	H1(2)	H3(1.9)			
1417395_at	*Klf4*	Kruppel-like factor 4 (gut)	H1(2.5)	H3(2.6)			
1417408_at	*F3*	coagulation factor III		H3(1.7)	R1(1.5)		
1418003_at	*1190002H23Rik*	RIKEN cDNA 1190002H23 gene		H3(1.5)	R1(1.5)		
1418059_at	*Eltd1*	EGF, latrophilin seven transmembrane domain containing 1				R3(2)	
1418595_at	*S3-12*	plasma membrane associated protein, S3-12					R6(1.9)
1419647_a_at	*Ier3*	immediate early response 3	H1(3.7)	H3(2.9)			
1419816_s_at	*Errfi1*	ERBB receptor feedback inhibitor 1		H3(1.7)	R1(1.5)		
1420088_at	*Nfkbia*	nuclear factor of kappa light polypeptide gene enhancer in B-cells inhibitor, alpha		H3(1.5)			
1420150_at	*Spsb1*	splA/ryanodine receptor domain and SOCS box containing 1		H3(1.7)	R1(2)		
1420376_a_at	*H3f3a /// H3f3b /// LOC100045490*	H3 histone, family 3A /// H3 histone, family 3B /// similar to H3 histone, family 3A	H1(1.2)	H3(1.4)	R1(1.5)	R3(1.3)	
1420772_a_at	*Tsc22d3*	TSC22 domain family, member 3				R3(1.5)	R6(1.3)
1420909_at	*Vegfa*	vascular endothelial growth factor A		H3(2)	R1(2)		

Supplementary Table S4.(Cont')

Probe sets	Gene Symbol	Gene Title	Time Point(fold of expression change)
1422818_at	*Nedd9*	neural precursor cell expressed, developmentally down-regulated gene 9	R1(1.7)
1423130_a_at	*Sfrs5*	splicing factor, arginine/serine-rich 5 (SRp40, HRS)	H1(1.4) H3(1.6) R1(1.7) R3(1.7)
1424638_at	*Cdkn1a*	cyclin-dependent kinase inhibitor 1A (P21)	R1(2.2)
1424671_at	*Plekhf1*	pleckstrin homology domain containing, family F (with FYVE domain) member 1	H3(1.7) R1(2)
1425281_a_at	*Tsc22d3*	TSC22 domain family, member 3	R3(1.6) R6(1.5)
1425631_at	*Ppp1r3c*	protein phosphatase 1, regulatory (inhibitor) subunit 3C	H3(1.6)
1425895_a_at	*Id1*	inhibitor of DNA binding 1	H1(1.6)
1426243_at	*Cth*	cystathionase (cystathionine gamma-lyase)	R3(2)
1426925_at	*Rc3h2*	ring finger and CCCH-type zinc finger domains 2	H1(1.5) H3(1.7) R1(1.7)
1428306_at	*Ddit4*	DNA-damage-inducible transcript 4	H1(2.5) H3(5.2) R1(3) R6(1.9)
1428352_at	*Arrdc2*	arrestin domain containing 2	R1(3.6)
1429050_at	*Chic2*	cysteine-rich hydrophobic domain 2	H3(1.8) R1(1.8)
1429456_a_at	*Polr3e*	polymerase (RNA) III (DNA directed) polypeptide E	H3(2) R1(1.7)
1429735_at	*1110003F05Rik*	RIKEN cDNA 1110003F05 gene	H1(1.7) H3(1.8)
1430295_at	*Gna13*	guanine nucleotide binding protein, alpha 13	R1(1.5) R3(1.5) R6(1.4)
1430357_at	*H3f3b*	H3 histone, family 3B	H1(6.9) H3(11)
1430798_x_at	*Mrpl15*	mitochondrial ribosomal protein L15	R1(2.1) R3(1.9)
1432006_at	*Ap2a2*	adaptor protein complex AP-2, alpha 2 subunit	H3(2.3) R1(1.8)
1432007_s_at	*Ap2a2*	adaptor protein complex AP-2, alpha 2 subunit	H1(1.9) H3(3) R1(2.3)
1433634_at	*Irf2bp2*	interferon regulatory factor 2 binding protein 2	H3(1.5) R1(1.3)
1433675_at	*Snhg1*	small nucleolar RNA host gene (non-protein coding) 1	H3(1.5)

Supplementary Table S4.(Cont')

**Probe sets**	Gene Symbol	Gene Title	**Time Point(fold of expression change)**					
1433837_at	*8430408G22Rik*	RIKEN cDNA 8430408G22 gene	H1(7.1)	H3(8.5)				
1434167_at	*Slc35e4*	solute carrier family 35, member E4		H3(1.5)	R1(1.6)	R3(1.2)		
1434350_at	*Axud1*	AXIN1 up-regulated 1			R1(1.8)			
1434817_s_at	*Rprd2*	Regulation of nuclear pre-mRNA domain containing 2			R1(4.9)	R3(5.2)		
1435188_at	*Gm129*	gene model 129, (NCBI)			R1(2.7)		R6(2.1)	
1436202_at	*Malat1*	metastasis associated lung adenocarcinoma transcript 1 (non-coding RNA)	H1(2.2)	H3(2.6)				
1437100_x_at	*Pim3*	proviral integration site 3		H3(1.4)	R1(1.7)			
1437132_x_at	*Nedd9*	neural precursor cell expressed, developmentally down-regulated gene 9			R1(1.6)			
1437313_x_at	*Hmgb2*	high mobility group box 2		H3(2)	R1(2.9)			
1438133_a_at	*Cyr61*	cysteine rich protein 61	H1(10)	H3(3.8)	R1(2)			
1438157_s_at	*Nfkbia*	nuclear factor of kappa light polypeptide gene enhancer in B-cells inhibitor, alpha	H1(1.7)	H3(2.4)	R1(1.7)		R6(1.5)	
1438427_at	*Fam120b*	family with sequence similarity 120, member B		H3(2.3)	R1(2.5)			
1440417_at	*D19Ertd409e*	DNA segment, Chr 19, ERATO Doi 409, expressed	H1(1.8)	H3(2.6)				
1441598_at	*Tmeff2*	transmembrane protein with EGF-like and two follistatin-like domains 2	H1(1.7)	H3(1.6)	R1(1.8)	R3(1.6)		
1441799_at	*6030422H21Rik*	RIKEN cDNA 6030422H21 gene		H3(4.4)	R1(2.9)			
1443471_at	*Zbtb20*	zinc finger and BTB domain containing 20		H3(2.6)				
1444107_at	*C130039016Rik*	RIKEN cDNA C130039016 gene			R1(2.1)			
1444564_at	*Apod /// LOC100047583*	apolipoprotein D /// similar to apolipoprotein D	H1(1.5)	H3(2.1)	R1(2.2)	R3(2.4)	R6(2.4)	R12(2.3)
1444681_at	*Erc2*	CAST1/ERC2 splicing variant-1 mRNA, complete cds, alternatively spliced	H1(2.5)	H3(3.8)	R1(5)	R3(5.1)	R6(3.4)	

Supplementary Table S4.(Cont')

Probe sets	Gene Symbol	Gene Title	Time Point(fold of expression change)				
1444722_at	---	---	H1(2)	H3(2.4)			
1444749_at	---	---	H1(4.4)	H3(7)			R6(2.1)
1446509_at	*Smox*	spermine oxidase		H3(2.3)			
1448181_at	*Klf15*	Kruppel-like factor 15		H3(1.6)	R1(2)		
1448231_at	*Fkbp5*	FK506 binding protein 5				R3(2.4)	R6(2.1)
1448272_at	*Btg2*	B-cell translocation gene 2, anti-proliferative	H1(2.7)				
1448306_at	*Nfkbia*	nuclear factor of kappa light polypeptide gene enhancer in B-cells inhibitor, alpha	H1(1.8)	H3(2.9)	R1(1.9)		R6(1.4)
1448397_at	*Gjb6*	gap junction protein, beta 6					R6(1.3)
1448890_at	*Klf2*	Kruppel-like factor 2 (lung)	H1(2.6)				
1449007_at	*Btg3 /// EG654432 /// LOC1000484[illegible]*	B-cell translocation gene 3 /// predicted gene, EG654432 /// similar to BTG3			R1(1.4)	R3(1.4)	
1449731_s_at	*Nfkbia*	nuclear factor of kappa light polypeptide gene enhancer in B-cells inhibitor, alpha	H1(1.5)	H3(2.3)	R1(1.5)		R6(1.5)
1451069_at	*Pim3*	proviral integration site 3	H1(1.3)	H3(1.6)	R1(1.9)		
1451959_a_at	*Vegfa*	vascular endothelial growth factor A		H3(1.7)	R1(1.5)		
1452414_s_at	*Ccdc86*	coiled-coil domain containing 86		H3(1.9)	R1(1.8)		
1452519_a_at	*Zfp36*	zinc finger protein 36	H1(2.6)	H3(2.1)			
1452534_a_at	*Hmgb2*	high mobility group box 2		H3(2)	R1(2.7)		
1453147_at	*Polr3e*	polymerase (RNA) III (DNA directed) polypeptide E		H3(1.8)	R1(1.8)	R3(1.5)	R6(1.6)
1454197_a_at	*Ccdc86*	coiled-coil domain containing 86		H3(1.5)	R1(1.4)		
1454617_at	*Arrdc3*	arrestin domain containing 3	H1(1.3)	H3(2.2)	R1(1.5)		R6(1.3)
1454685_at	*Gpr146*	G protein-coupled receptor 146		H3(1.5)	R1(1.8)		
1455725_a_at	*H3f3a /// H3f3b /// LOC100045490*	H3 histone, family 3A /// H3 histone, family 3B /// similar to H3 histone, family 3A		H3(1.3)	R1(1.3)		
1456159_at	---	---	H1(2.5)	H3(3.3)			
1456347_at	---	---	H1(1.9)	H3(1.7)			
1458508_at	*Matr3*	matrin 3	H1(1.8)	H3(2.1)			
1459548_at	*Spire1*	spire homolog 1 (Drosophila)		H3(1.9)			

Supplementary Table S5 Common genes regulated by both hypoxia preconditioning(HP) and acute ischemia in the brain of either RAt MCAO model or Mouse MCAO model.

**Gene Symbol**	**Gene Title**	**Regulation After HP (within 24hr)**	**Regulation After MCAO(within 24hr)***	**Probe Set ID**	**GeneBank ID**
Ier3	immediate early response 3	Up	Up (Rat & Mice)	1419647_a_at	NM_133662
Sfrs5	splicing factor, arginine/serine-rich 5 (SRp40, HRS)	Up	Up (Mice)	1423130_a_at	AW212917
Mt2	metallothionein 2	Up	Up (Mice)	1428942_at	AA796766
Gadd45b	growth arrest and DNA-damage-inducible 45 beta	Up	Up (Mice)	1449773_s_at, 1450971_at	AI323528, AK010420
Ctla2a /// Ctla2b	cytotoxic T lymphocyte-associated protein 2 alpha /// cytotoxic T lymphocyte-associated protein 2 beta	Up	Up (Mice)	1416811_s_at	NM_007796
Cebpd	CCAAT/enhancer binding protein (C/EBP), delta	Up	Up (Mice)	1423233_at	BB831146
Cdkn1a	cyclin-dependent kinase inhibitor 1A (P21)	Up	Up (Mice)	1424638_at	AK007630
Zfp36	zinc finger protein 36	Up	Up (Rat)	1452519_a_at	X14678
Jun	Jun oncogene	Up	Up (Rat)	1417409_at	NM_010591
Errfi1	ERBB receptor feedback inhibitor 1	Up	Up (Rat)	1416129_at, 1419816_s_at	NM_133753 ,AI788755
Cth	cystathionase (cystathionine gamma-lyase)	Up	Up (Rat)	1426243_at	BC019483
Btg2	B-cell translocation gene 2, anti-proliferative	Up	Up (Rat)	1416250_at,1448272_at	NM_007570
Adm	adrenomedullin	Up	Up (Rat)	1416077_at,1447839_x_at	NM_009627 ,AV378441
(unknown EST)	(unknown EST)	Up(R1& R6) then Down(R12-24)	Down (Mice)	1435119_at	BE956710
(unknown EST)	(unknown EST)	Down	Down (Mice)	1456864_at	BB210733
AU041133	expressed sequence AU041133	Down	Down (Mice)	1444041_at	AU041133
Casp8ap2	caspase 8 associated protein 2	Down	Down (Mice)	1449217_at	NM_011997

Supplementary Table S5 (cont')

Gene Symbol	Gene Title	Regulation After HP (within 24hr)	Regulation After MCAO(within 24hr)*	Probe Set ID	GeneBank ID
Rpa3	replication protein A3	Down	Down (Mice)	1448938_at	NM_026632
Neurod1	neurogenic differentiation 1	Down	Down (Rat)	1426412_at	BM116592
Sult1a1	sulfotransferase family 1A, phenol-preferring, member 1	Down(H1)then Up(R6)	Down (Rat)	1427345_a_at	AK002700

* Mouse data were from Sarabi et al, 2008; Rat data were from Lu et al, 2002.
